# Canopy and leaf vein segmentation and topology-guided localization of stem points in tobacco plants using YOLO-ODS

**DOI:** 10.3389/fpls.2026.1930225

**Published:** 2026-10-07

**Authors:** Jiaxing Qing, Donghao Li, Xiwen Luo, Zhihao Zeng, Jinhong Chen, Yawen Liu, Jingqi Xu, Shan Zeng, Yukun Yang, Yu Wang

**Affiliations:** 1 College of Engineering, South China Agricultural University, Guangzhou, China; 2 Key Lab of Key Technology for South Agricultural Machine and Equipment, Ministry of Education, P.R. China (KTSAME), Guangzhou, China; 3 College of Food Science, South China Agricultural University, Guangzhou, China; 4 School of Information Technology and Engineering, Guangzhou College of Commerce, Guangzhou, China

**Keywords:** instance segmentation, leaf vein topology, nadir-view UAV imagery, stem point localization, tobacco plant, YOLO-ODS

## Abstract

Accurate stem point localization is critical for intelligent tobacco production but remains challenging in low-altitude nadir-view UAV imagery. This is because the visible upper stem after topping is small, weakly textured, and visually similar to surrounding canopy tissues. This study formulates stem point localization as a topology-guided geometric inference problem based on canopy and leaf vein structures. A lightweight instance segmentation model, YOLO-ODS, was developed on YOLOv11n-Seg to jointly segment tobacco canopies and leaf veins from UAV imagery. The model integrates an Orientation-Aware Convolution Module (OACM), a Dual Interactive Attention Module (DIAM), and Slide Loss to enhance directional feature representation and improve discrimination of fine-grained structures. Based on segmentation results, leaf vein skeletons are extracted and local trend lines are fitted to estimate stem points via geometric convergence under canopy constraints. Experimental results showed that YOLO-ODS achieved a Recall of 86.79%, a mask mAP@0.5 of 90.19%, and a mask mAP@0.5:0.95 of 67.20%, outperforming the YOLOv11n-Seg baseline by 2.20, 1.14, and 2.10 percentage points, respectively. For the leaf vein category, the mask mAP@0.5:0.95 reached 51.49%. With 2.86 M parameters and 10.19 GFLOPs, YOLO-ODS remained lightweight for field deployment. The proposed localization method achieved an *MAE* of 12.05 px and an *RMSE* of 12.99 px, reducing *MAE* and *RMSE* by 51.2% and 52.6%, respectively, compared with YOLOv11n-pose. These results suggest that integrating lightweight instance segmentation with leaf vein topology may provide a promising approach for improving stem point localization under complex field conditions.

## 1 Introduction

Tobacco is an important economic crop with substantial agricultural and industrial value (Banožić et al., 2020; Shen et al., 2024). In addition to its role in agricultural production, tobacco and its processing residues contain cellulose, alkaloids, solanesol, and other valuable compounds with potential applications in the energy, pharmaceutical, and chemical industries (Tita et al., 2020; Duan et al., 2026; Xu et al., 2025; Lu et al., 2026). Despite the rapid development of agricultural mechanization, several key tobacco production operations, including harvesting and precision field management, still rely heavily on manual labor (Zhao et al., 2026). Consequently, the development of intelligent tobacco production equipment has become an important research objective. Stem localization is a fundamental prerequisite for many intelligent tobacco production tasks. For example, robotic harvesting relies on plant spatial coordinates for navigation and operation planning, and precision field management often uses individual plants as basic operational units. Therefore, robust stem localization is essential for improving the efficiency and automation level of tobacco production systems.

Existing studies related to tobacco stem localization can generally be divided into aerial-image-based (Nan et al., 2026; Liu et al., 2025a) and ground-based approaches (Yang et al., 2023). Aerial-image-based methods typically utilize UAV imagery to identify tobacco plants through canopy detection and phenotypic feature extraction (Shahid et al., 2024; Lin et al., 2023). In these methods, stem positions are often approximately estimated using geometric features such as canopy centroids or plant centers. However, tobacco plants often exhibit asymmetric leaf growth, canopy deformation, and plant inclination, causing the geometric center of the canopy to deviate from the actual stem position. Ground-based approaches directly observe stem structures using close-range imaging or three-dimensional point clouds. Previous studies have achieved accurate segmentation of stems and other plant organs from ground-view images and point clouds (Bi et al., 2026; Liu et al., 2025b). Nevertheless, dense foliage frequently occludes stem tissues, reducing stem visibility and limiting localization robustness under practical field conditions.

For intelligent field operations, it is not always necessary to recover the complete stem structure. Instead, a representative stem-related coordinate is often sufficient for harvesting, navigation, plant-level localization, and precision management (Lai et al., 2026; Wu et al., 2026). After topping (Gu et al., 2023), the removal of the apical bud exposes part of the upper stem region, providing visual and morphological cues for stem localization from low-altitude nadir-view imagery. In this study, the pixel centroid of the visible upper stem region is defined as the stem point, as shown in **Figure 1**.

**Figure 1:**
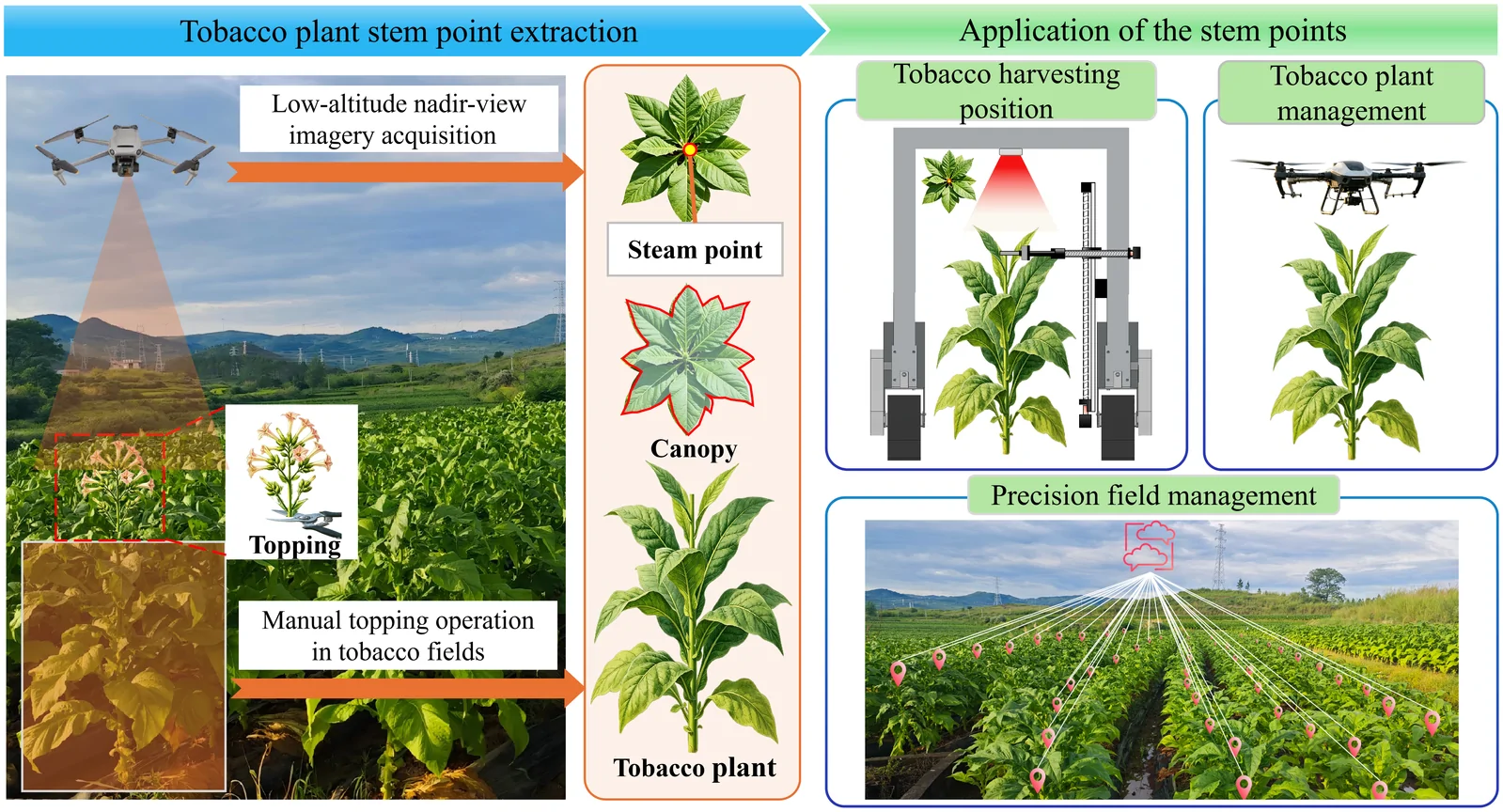
Definition of the stem point in topped tobacco plants and its application in intelligent field operations.

Recent advances in deep learning have significantly improved keypoint localization in agricultural vision tasks. Object detection methods estimate target positions through bounding boxes and have been widely applied in harvesting and plant organ localization tasks (Li et al., 2025; Yang et al., 2023). However, localization precision is constrained by the coarse spatial representation of bounding boxes. Keypoint detection methods directly regress target coordinates and generally provide higher localization accuracy (Zhang et al., 2026; Yi et al., 2026), but their performance often deteriorates when target points are extremely small and visually weak. For tobacco stem point localization in low-altitude nadir-view imagery, the stem point itself lacks distinctive visual texture. Therefore, direct keypoint regression based only on local appearance features is prone to instability under complex field conditions.

Compared with direct object detection and keypoint regression, structural analysis based on plant-organ segmentation provides richer morphological information and enables the explicit use of organ topology, spatial continuity, and geometric constraints (Chen et al., 2025). Such structural priors are particularly useful for localizing biologically meaningful keypoints that are visually weak or difficult to distinguish from surrounding tissues (Wang et al., 2025). Previous studies have incorporated plant morphology and spatial relationships into phenotypic analysis and keypoint localization. Wu et al. (2019) reconstructed maize skeletons from three-dimensional point clouds to extract structural information, while He et al. (2024) used the morphological and growth-direction characteristics of soybean pods for keypoint analysis. Li et al. (2025) and Deng et al. (2025) segmented fruits and peduncles and subsequently determined harvesting or cutting points according to their spatial topological relationships. These studies demonstrate that structural and geometric priors can improve localization robustness compared with appearance-based prediction alone.

Tobacco morphology provides a suitable biological basis for topology-guided stem point localization (Ma et al., 2026; Xu et al., 2024). Tobacco leaves originate from the stem region and generally extend radially outward, while their primary veins follow the main leaf-growth directions and maintain strong directional continuity. Therefore, the inward extensions of multiple primary leaf veins tend to converge near the stem region. Compared with the exposed upper stem, which is small and weakly textured in nadir-view UAV imagery, primary leaf veins provide clearer and more stable directional information.

Although topology-assisted localization has been explored in plant phenotyping and harvesting applications, existing methods mainly rely on organ morphology, spatial adjacency, or the relationship between fruits and peduncles. The geometric convergence of multiple leaf vein directions has rarely been used to infer stem points from aerial images. Therefore, this study formulates tobacco stem point localization as a topology-guided geometric inference problem. leaf vein directions are extracted under canopy constraints, and their geometric convergence is used to estimate the stem position. This approach transforms the localization of a visually weak stem point into an interpretable inference process based on biologically meaningful leaf vein topology.

Based on these observations, this study proposes a topology-guided stem point localization framework for low-altitude nadir-view UAV imagery of tobacco plants. The main contributions of this study are summarized as follows:

1. A tobacco canopy and leaf vein instance segmentation dataset was constructed using low-altitude UAV imagery collected under diverse field and illumination conditions. The dataset supports canopy structural analysis and topology-guided stem point localization in tobacco plants.
2. A lightweight instance segmentation model, named YOLO with Orientation-aware convolution, Dual interactive attention, and Slide Loss (Yu et al., 2024) (YOLO-ODS), was developed based on YOLOv11n-Seg. By incorporating OACM, DIAM, and Slide Loss, the model enhances the segmentation of tobacco canopies and slender leaf vein structures while maintaining low computational complexity.
3. A topology-guided stem point localization method was proposed by combining canopy constraints and leaf vein geometric topology. The method uses leaf vein skeleton extraction, local trend-line fitting, and convergence-based candidate optimization to transform weak stem point detection into an interpretable geometric inference process.

## 2 Materials and methods

### 2.1 Dataset construction and annotation

Data were collected from tobacco fields located in Jiaoling County, Meizhou City, Guangdong Province, China (24.658963° N, 116.171477° E), and Xingzi Town, Qingyuan City, Guangdong Province, China (24.997284° N, 112.559461° E). The image acquisition periods were May 25-27, 2025, and June 29-July 1, 2025. The tobacco variety cultivated in the experimental fields was Yunyan 87, which is widely planted in South China. A DJI Mavic 3 UAV was used to acquire low-altitude nadir-view RGB images at a flight height of approximately 5–8 m above the ground. To improve the representativeness of the dataset under different field conditions, image acquisition was conducted under different illumination conditions, including sunny, cloudy, and overcast weather. During data acquisition, the boundaries of previously collected field regions were manually recorded. The flight areas were adjusted during subsequent acquisitions to avoid repeated imaging of the same field regions, ensuring that different original UAV images did not contain overlapping field scenes. Finally, a total of 773 original UAV images with an image resolution of 5280 × 3956 pixels were collected.

To minimize the risk of data leakage caused by assigning patches derived from the same source image to different subsets, dataset partitioning was performed at the source-image level before patch extraction. Thus, all patches derived from a given source image were retained within the same subset. After geometric correction, the 773 original UAV images were divided into training, validation, and test subsets according to an approximate ratio of 7:2:1. The three subsets contained 540, 154, and 79 original UAV images, respectively. The original images in each subset were independently cropped into 1280 × 1280-pixel patches using a non-overlapping sliding-window strategy. No overlap was introduced between adjacent patches during cropping. Because the original image dimensions were not exact multiples of 1280 pixels, boundary regions smaller than the predefined patch size were discarded rather than padded or resized. Each original UAV image generated 12 non-overlapping image patches, resulting in 9,276 cropped patches. The cropped patches within each subset were then screened independently. Only patches containing at least one complete tobacco plant were retained for subsequent annotation. A total of 2,634 valid image patches were obtained from the three subsets. After quality inspection, patches containing severe blur, incomplete plant structures, or invalid annotation regions were removed. Finally, 2,608 image patches were retained, including 1,825 training image patches, 522 validation image patches, and 261 test image patches.

To further assess the robustness of the dataset partitioning strategy, an additional acquisition-sequence-based independent split was constructed as described in **Supplementary Material** Section 1. Under this strategy, all images acquired during the same UAV flight sequence, together with their derived image patches, were assigned exclusively to a single subset. The detailed experimental design and dataset partitioning scheme are provided in **Supplementary Table S1**. This stricter partitioning strategy reduced the possibility that visually similar images originating from the same acquisition sequence appeared in both the training and evaluation sets. The corresponding evaluation results are reported in **Supplementary Table S2**. The results demonstrated that YOLO-ODS maintained highly consistent segmentation performance under both the source-image-level partitioning strategy used in the main manuscript and the stricter acquisition-sequence-based partitioning strategy, indicating the robustness of the proposed model against potential data similarity bias caused by UAV image acquisition conditions.

Tobacco canopies and primary leaf veins were manually annotated using X-AnyLabeling-CPU with polygon masks. Two instance categories were defined: canopy and leaf vein. Each tobacco canopy was annotated as an independent canopy instance, and each visible primary leaf vein was annotated as an independent leaf vein instance. Secondary veins, severely occluded veins, and unclear or disconnected vein fragments were excluded from annotation.

The statistical composition of the constructed dataset is summarized in **Table 1**. All statistical analyses were conducted before data augmentation. The number of leaf veins per canopy represents the average number of annotated primary leaf vein instances associated with each canopy instance. Pixel proportions were calculated as the ratio of pixels covered by the corresponding annotation masks to the total number of image pixels. As shown in **Table 1**, the annotated dataset comprised 2,608 original image patches, including 3,622 canopy instances and 26,183 independently annotated primary leaf vein instances. The dataset covered diverse illumination conditions, including 950 sunny images, 855 cloudy images, and 803 overcast images. All three illumination conditions were represented in the training, validation, and test subsets. At the pixel level, canopy regions accounted for approximately 35.73% of the total image area, whereas leaf vein regions accounted for only 1.40%. This difference indicates a significant pixel-level imbalance between large canopy regions and slender leaf vein structures. The small proportion of leaf vein pixels also highlights their fine-scale characteristics and potential segmentation challenges.

**Table 1:** Statistical composition of the tobacco canopy and leaf vein instance segmentation dataset.

Dataset subset	Number of images	Sunny images	Cloudy images	Overcast images	Canopy instances	leaf vein instances	Leaf veins per canopy	Canopy pixel proportion (%)	leaf vein pixel proportion (%)
Training set	1825	706	615	504	2531	18183	7.18	35.42	1.38
Validation set	522	172	149	201	727	5146	7.08	36.20	1.42
Test set	261	72	91	98	364	2854	7.84	36.08	1.43
Overall	2608	950	855	803	3622	26183	7.23	35.73	1.4

To improve model generalization to variations in illumination, image quality, and field conditions, data augmentation was applied exclusively to the training subset. Four augmentation operations—brightness adjustment, Gaussian blur, image rotation, and Gaussian noise—were randomly applied to the training images (Li et al., 2025). For each original training patch, one augmented counterpart was generated while the original image was retained, thereby increasing the training set from 1,825 to 3,650 images. The detailed augmentation settings are summarized in **Table 2**. For each original image, we generated one augmented sample using a random combination of four operations based on their nominal sampling probabilities, with the constraint that at least one operation was always applied. When multiple augmentation operations were selected, they were applied sequentially in the fixed order of rotation first, followed by brightness adjustment, then Gaussian noise, and finally Gaussian blur. If no augmentation operation was selected, the sampling process was repeated to ensure that each generated image contained at least one transformation. All augmentations were performed with a fixed random seed (42) to ensure reproducibility. The augmented images were used exclusively for model training and were not included in the validation subset, test subset, or dataset statistical analysis.

**Table 2:** Parameters of data augmentation strategies applied to the training dataset.

Augmentation operation	Parameter setting	Nominal sampling probability	Purpose
Brightness adjustment	Brightness factor randomly sampled from 0.8 to 1.2	0.5	Simulate illumination variations under different field conditions
Gaussian blur	Kernel size randomly selected from 3 × 3 or 5 × 5	0.5	Simulate mild defocus and reduced image sharpness
Image rotation	Random rotation angle from -15° to 15°; polygon annotations transformed synchronously	0.3	Improve robustness to image orientation variations
Gaussian noise	Standard deviation randomly sampled from 0.01 to 0.05 on a [0, 1]-normalized pixel scale	0.3	Simulate sensor noise and UAV imaging disturbances

To further investigate the effects of individual data augmentation strategies on model performance and robustness, additional ablation experiments were conducted in **Supplementary Material** Section 2. The quantitative comparisons are reported in **Supplementary Tables S3** and **S4**, and the corresponding qualitative visualization results are presented in **Supplementary Figure S1**.

Finally, the final training dataset consisted of 3,650 image patches, including 1,825 original training patches and 1,825 augmented counterparts. Together with the validation set (522 patches) and test set (261 patches), a total of 4,433 image patches were used for model development and evaluation. The annotations were subsequently converted into the YOLO instance segmentation format to establish the tobacco canopy and leaf vein segmentation dataset. The overall dataset construction process is illustrated in **Figure 2**.

**Figure 2:**
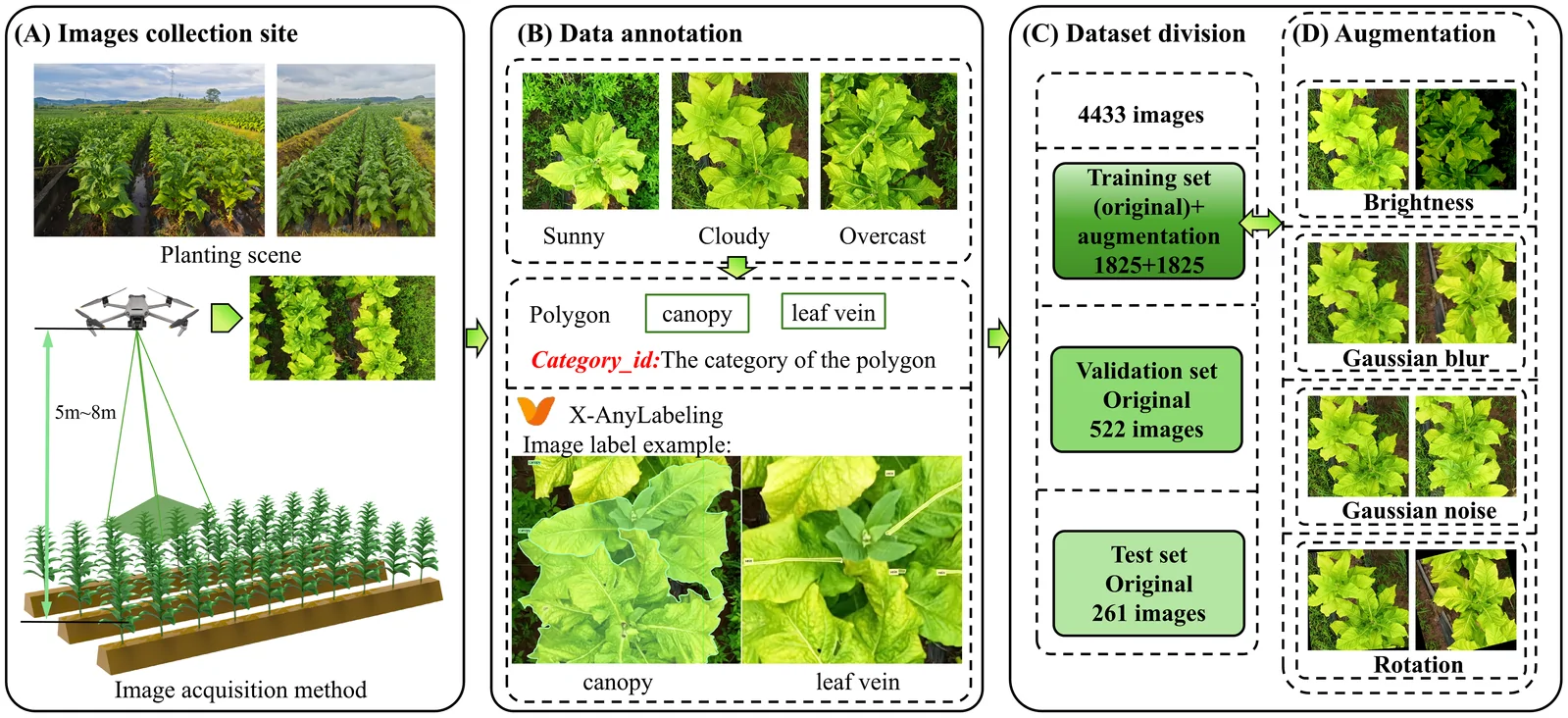
Overview of the dataset construction and annotation pipeline for tobacco canopy and leaf vein instance segmentation. **(A)** Locations and procedures of UAV image acquisition; **(B)** Dataset construction and annotation workflow; **(C)** Dataset splitting into training, validation, and test sets; **(D)** Image augmentation strategies applied to the training dataset.

### 2.2 YOLO-ODS framework

YOLOv11n is a lightweight model in the Ultralytics YOLOv11 series. It features a compact parameter design, high inference efficiency, and low deployment overhead, making it suitable for UAV edge devices and intelligent mobile terminals (Zhu et al., 2024). YOLOv11n-Seg extends the YOLOv11n detection framework with an instance segmentation head, enabling simultaneous prediction of object categories, bounding boxes, and pixel-level masks. The network mainly consists of a Backbone, a Neck, and a Head (Khanam and Hussain, 2024). The Backbone extracts multi-scale image representations, the Neck aggregates semantic features across different resolutions, and the Head produces instance segmentation outputs.

In low-altitude nadir-view images, tobacco plants are often densely distributed, and their canopies may overlap with each other. Leaf veins are slender, low-contrast structures and occupy only a small proportion of image pixels, which makes accurate instance segmentation difficult. When YOLOv11n-Seg is directly applied to this task, problems such as missed leaf vein detection, incomplete canopy boundary segmentation, and confusion between adjacent plant regions may occur. These errors can further affect the accuracy of subsequent stem point localization. Therefore, this study proposes YOLO-ODS, a lightweight tobacco canopy and leaf vein segmentation model based on YOLOv11n-Seg. The structure of YOLO-ODS is shown in **Figure 3**.

**Figure 3:**
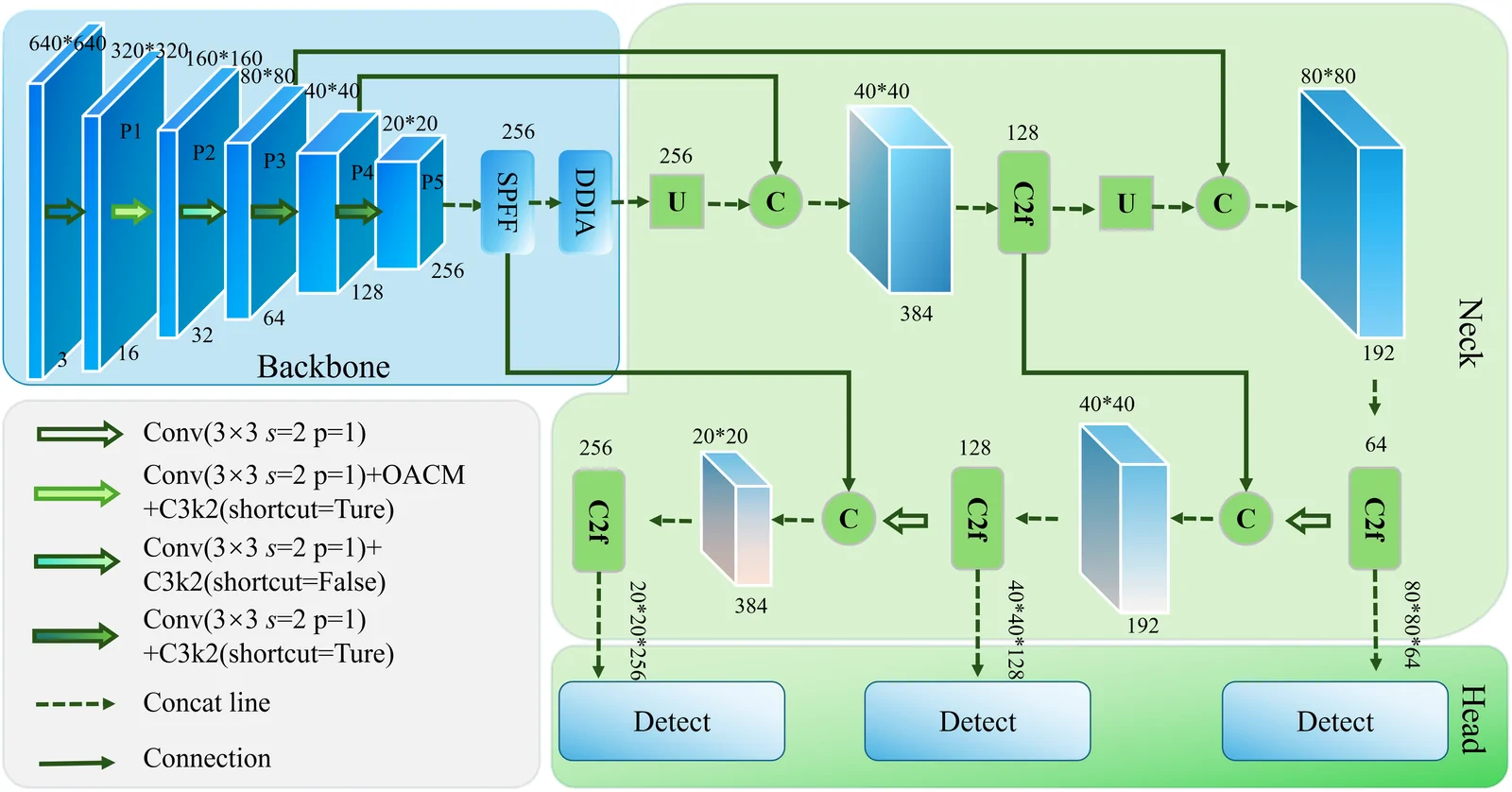
Overall architecture of the proposed YOLO-ODS model.

YOLO-ODS makes three main improvements to the baseline model. First, an Orientation-Aware Convolution Module (OACM) is introduced into the shallow feature extraction stage of the Backbone. Multi-directional convolution branches are used to enhance the representation of directional structures, such as leaf veins, leaf edges, and canopy boundaries. Second, a Dual Interactive Attention Module (DIAM) is introduced to jointly model spatial attention and channel attention, allowing the model to emphasize key regions and important semantic channels while suppressing field background noise. Third, Slide Loss is adopted to dynamically adjust the loss contribution of samples with different matching qualities, thereby improving the model’s learning ability for fine-grained targets such as leaf veins.

These three components are designed to address complementary challenges: OACM enhances directional feature modeling for slender structures, DIAM improves feature selection under complex backgrounds, and Slide Loss mitigates imbalance between canopy and leaf vein instances during optimization.

#### 2.2.1 Orientation-aware convolution module

Leaf veins often exhibit slender and multi-directional structural features. Conventional convolution lacks explicit directional modeling ability and may not effectively capture the continuity and edge consistency of these structures. To enhance the model’s ability to represent multi-directional fine-grained features, this study proposes an OACM, as shown in **Figure 4**.

**Figure 4:**
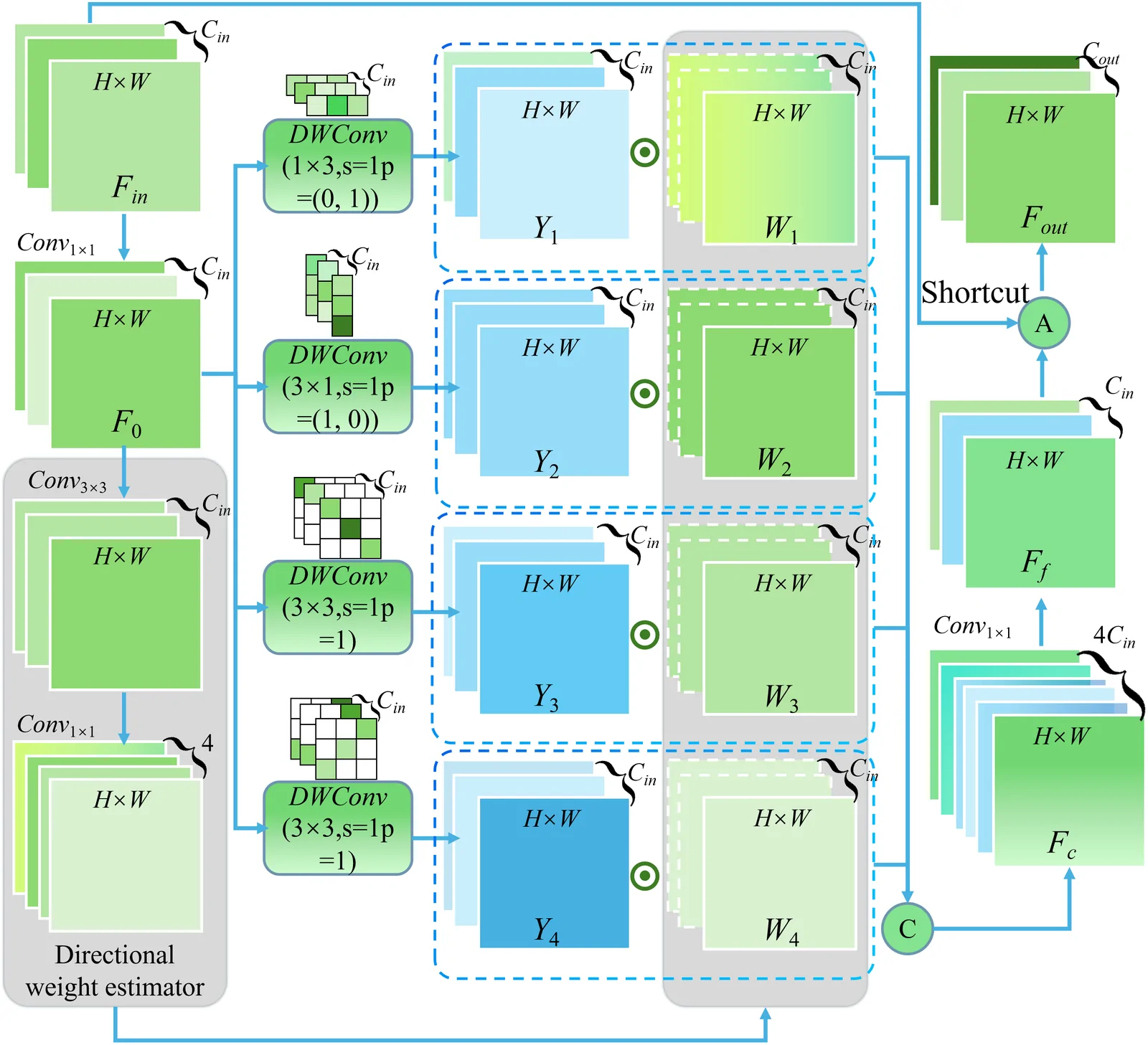
Structure of the orientation-aware convolution module.

Let the input feature map be denoted as $F_{in}\in R^{B\times C_{in}\times H\times W}$, and the output feature map be denoted as $F_{out}\in R^{B\times C_{out}\times H\times W}$, where *B*, *H*, and *W* denote the batch size, height, and width of the feature map, respectively, and C_in_ and C*_out_* denote the numbers of input and output channels. In OACM, the input and output channel numbers are kept consistent.

*F_in_* is first processed by a 1 × 1 convolution for channel alignment and linear mapping, producing an intermediate feature map *F*_0_. Then, *F*_0_ is fed into four depthwise separable convolution branches corresponding to four directions. The convolution kernels are 1 × 3, 3 × 1, 3 × 3, and 3 × 3, which are used to extract horizontal, vertical, 45°, and 135° directional features, respectively. The outputs of the four branches are denoted as Y_d_ (d = 1, 2, 3, 4).

In order to enhance the direction sensitivity of 45° and 135° branches, explicit directional priors are introduced during convolution kernel initialization. The initialization strategy is empirically designed to bias early training toward directional sensitivity while allowing adaptive refinement during optimization. For the 45° branch, positions close to the main diagonal of the 3 × 3 kernel are assigned higher initial weights. The initialization rule is defined as:


$$w_{1}\left(i,j\right)=\{\begin{array}{c} \mu +0.5\sigma \;\left|i-j\right|\leq 1\\ \mu -0.1\text{σ}\;\left|i-j\right|>1 \end{array}$$

where *w*_1_(*i*, *j*) represents the weight of the 45° branch convolution kernel at the position of the *i*-th row and the *j*-th column; *μ* is the mean value of the convolution kernel; σ is the standard deviation of convolution kernel.

For the 135° branch, positions close to the anti-diagonal of the 3 × 3 kernel are assigned higher initial weights. When zero-based indexing is used, the initialization rule can be defined as:


$$w_{2}\left(i,j\right)=\{\begin{array}{c} \mu +0.5\sigma \;\left|\left(i+j\right)-2\right|\leq 1\\ \mu -0.1\sigma \;\left|\left(i+j\right)-2\right|>1 \end{array}$$

Where *w*_2_(*i*, *j*) denotes the weight of the 135° branch convolution kernel at the position of the *i*-th row and the *j*-th column.

OACM further introduces a lightweight directional weight estimator composed of a 3 × 3 convolution, a 1 × 1 convolution, and a Sigmoid activation function. The intermediate feature map *F*_0_ is passed through the estimator to generate a set of spatial weight maps $W_{d}\in R^{B\times 1\times H\times W}(d=1,\;2,\;3,\;4)$ for the four directional branches. The *d*-th channel of W, denoted as (W_d_), represents the spatial response weight of the corresponding directional branch and characterizes the importance of different directional structures at each spatial position.

Using tensor broadcasting, each single-channel spatial weight map is multiplied with the corresponding directional feature map Y_d_ in a pixel-wise manner. The weighted multi-directional features are concatenated along the channel dimension to obtain the fused feature map $F_{C}\in {\text{R}}^{{B\times 4C}_{in}\text{×H×W}}$. A 1 × 1 convolution is then used for channel compression and information integration, producing the final fused feature $F_{f}\in {\text{R}}^{{\text{B×C}}_{in}\text{×H×W}}$. Finally, a residual connection is used to combine *F_f_* with the input feature *F_in_*, generating the output feature map *F_out_*. This residual design improves gradient propagation stability and preserves the original semantic information.

Through this design, OACM explicitly models and adaptively fuses multi-directional structural features while maintaining lightweight computational overhead. As a result, it improves the feature representation ability of the model in dense, overlapping, and fine-grained structural scenes.

#### 2.2.2 Dual interactive attention module

In complex field instance segmentation tasks, different channels and spatial regions of a feature map contribute unequally to the final segmentation results. To enhance the model’s ability to extract key canopy and leaf vein features while reducing interference from soil, weeds, shadows, and adjacent plants, this study proposes a Dual Interactive Attention Module (DIAM). DIAM generates spatial attention masks and channel attention weights in parallel and recalibrates the input features to improve feature discrimination. The processing flow of DIAM is shown in **Figure 5** and consists of three stages: a spatial attention branch, a channel attention branch, and a dual-attention fusion stage.

**Figure 5:**
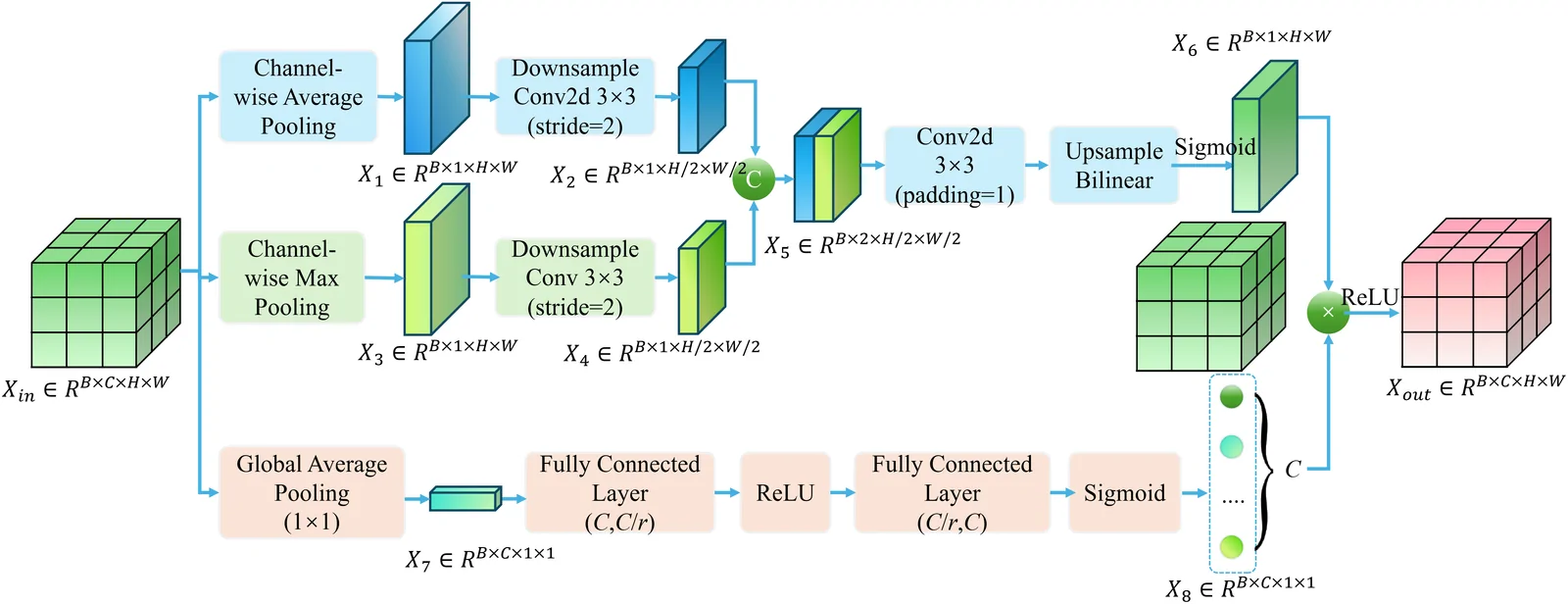
Structure of the dual interactive attention module.

In the spatial attention branch, the input feature map is denoted as ${\text{X}}_{\text{in}}\in {\text{R}}^{\text{B×C×H×W}}$. Global average pooling and global max pooling are first performed along the channel dimension to obtain two spatial descriptor maps, which characterize the response intensity of different image regions. The two descriptor maps are then downsampled using 3 × 3 convolutions to obtain compact spatial features. These features are concatenated along the channel dimension and further fused by convolution. Finally, the fused feature is restored to the same spatial size as the input feature map through a 3 × 3 convolution and bilinear upsampling. A *Sigmoid* activation function is then used to generate the spatial attention mask ${\text{X}}_{6}\in {\text{R}}^{B\times 1\times H\times W}$.

In the channel attention branch, global average pooling is first applied to the input feature map to obtain a global channel descriptor ${\text{X}}_{7}\in {\text{R}}^{B\times C\times 1\times 1}$. The descriptor is then passed through a two-layer fully connected network with *ReLU* activation in the middle. A Sigmoid activation function is used to constrain the channel weights to the range of [0,1], producing the channel attention mask ${\text{X}}_{8}\in {\text{R}}^{B\times C\times 1\times 1}$.

In the dual-attention fusion stage, the spatial attention mask and channel attention mask are applied to the original input feature through element-wise multiplication. The recalibrated feature is then activated by *ReLU* activation to obtain the output feature ${\text{X}}_{\text{out}}\in {\text{R}}^{\text{B×C×H×W}}$. By simultaneously highlighting important spatial regions and semantic channels, DIAM improves the robustness of the model in complex backgrounds and small-target segmentation scenarios.

#### 2.2.3 Slide loss

In tobacco canopy and leaf vein instance segmentation, there is a significant imbalance between categories and sample difficulties. The canopy usually occupies a larger area, has a more complete structure, and presents clearer boundaries, whereas leaf veins are slender, small in scale, and easily confused with background textures. During the training of YOLOv11n-Seg, conventional loss functions lack dynamic adjustment for samples with different matching qualities. As a result, the model may focus more on easily segmented samples and insufficiently learn fine-grained targets such as leaf veins.

To alleviate this problem, the Slide Loss strategy proposed by Yu et al. (2024) was adopted in this study. Without increasing model parameters or changing the network structure, Slide Loss adaptively modulates the loss weights of samples with different matching qualities through a piecewise dynamic weighting mechanism. Specifically, the dynamic threshold δ is calculated based on the average IoU of positive samples in the current training batch, and its lower bound is set to 0.2 to avoid unstable weight distribution in the early training stage. Based on δ, sample quality is divided into different intervals, and the weight function is defined as follows:


$$F(x)=\left\{ \begin{array}{l} 1\;x\leq \delta -0.1\;\\ e^{1-\delta \;}\;\delta -0.1<x<\delta \\ e^{1-x}\;x>\delta \end{array}\right.$$

where *x* denotes the matching quality, represented by IoU, and δ denotes the dynamic threshold.

According to this weighting function, when the sample matching quality is low ( x≤δ−0.1), the loss weight remains 1, preventing extremely low-quality samples from excessively interfering with gradient updates. When a sample falls within the critical transition interval ( δ−0.1<x<δ), it is assigned an enhanced weight of e^1-^δ, which strengthens the model’s learning of difficult boundary samples. When the matching quality is high (x > δ), the weight gradually decreases as the matching quality increases, thereby reducing the dominance of high-quality easy samples in the overall optimization direction.

Through this sliding piecewise modulation strategy, Slide Loss adaptively adjusts the gradient contribution according to sample quality during training. This allows the model to focus more on fine-grained structural targets such as leaf veins while maintaining convergence stability.

### 2.3 Stem point localization

#### 2.3.1 Stem point localization method

Based on the morphological characteristics of tobacco canopies, this study proposed a topology-guided stem point localization method. Instead of relying on weak stem appearance features, it infers the stem position from leaf vein convergence under canopy constraints. This improves both localization stability and the interpretability of the process. The method combines instance segmentation with geometric topology analysis. The complete workflow is shown in **Figure 6** and consists of the following main steps: instance segmentation with canopy-leaf vein association, leaf vein skeleton extraction and endpoint analysis, trend-line fitting on proximal leaf vein skeleton, candidate stem point construction and constraint filtering, and candidate stem point filtering.

**Figure 6:**
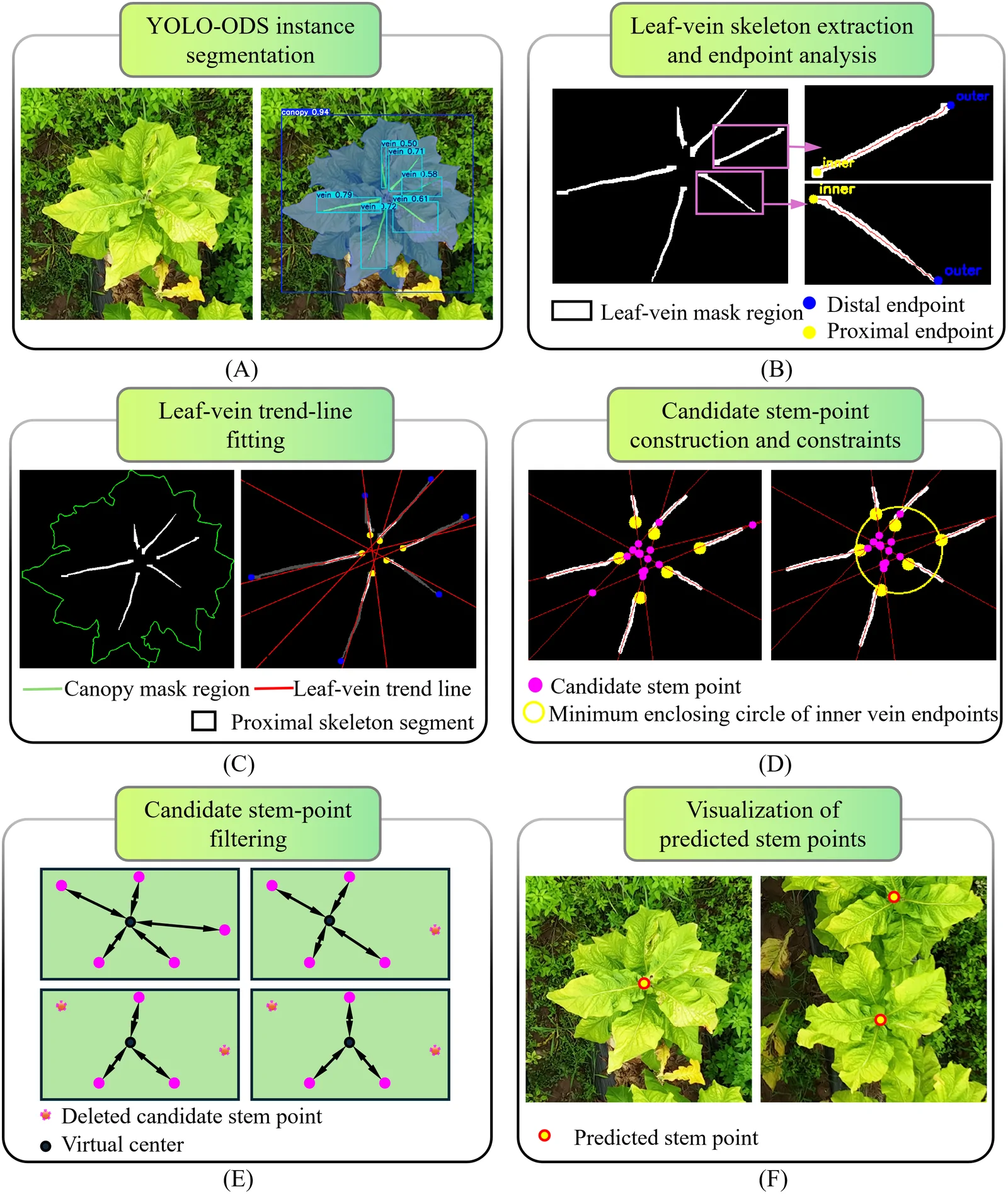
Workflow of topology-guided stem point localization based on canopy-leaf vein geometric relationships. **(A)** YOLO-ODS instance segmentation with canopy-leaf vein association; **(B)** leaf vein skeleton extraction and endpoint analysis; **(C)** Trend-line fitting on proximal leaf vein skeleton; **(D)** Candidate stem point construction and constraint filtering; **(E)** Candidate stem point filtering; **(F)** Visualization of predicted stem points.

First, the input UAV image was processed by YOLO-ODS to obtain pixel-level instance segmentation results for tobacco canopies and leaf veins (**Figure 6A**). Each predicted instance was represented by a pixel-level mask and a polygonal contour. According to the predicted category labels, all instances are divided into a canopy set and a leaf vein set. Because a single image may contain multiple tobacco canopies and multiple leaf veins, the spatial correspondence between canopies and leaf veins must be established before stem point localization. For this purpose, the geometric centroid of each leaf vein instance is calculated, and its spatial relationship with each canopy polygon is determined. If the centroid of a leaf vein lies inside a canopy polygon, the corresponding leaf vein is assigned to that canopy. After this step, a one-to-many mapping relationship was constructed between each tobacco canopy and its associated internal leaf veins.

For each canopy-associated leaf vein instance, the vein mask was first intersected with the corresponding canopy mask to exclude regions outside the target canopy. The resulting mask was refined using hole filling, morphological opening and closing, and small-object removal. Connected-component analysis was then performed, retaining only the largest component for subsequent processing. Skeletonization was applied to convert each refined leaf vein mask into a single-pixel-wide centerline while preserving its topology and main orientation (**Figure 6B**). Skeleton endpoints were identified as pixels with only one neighboring skeleton pixel. For each skeleton, the two principal endpoints were used to infer vein direction. Specifically, Euclidean distances between each endpoint and the canopy centroid were computed, and the endpoint closer to the centroid was defined as the proximal endpoint, while the other was defined as the distal endpoint (**Figure 6B**). Here, the canopy centroid serves only as a coarse geometric reference for determining the approximate inward direction of the leaf vein, rather than as an estimate of the stem position.

After identifying the proximal and distal endpoints of each valid leaf vein skeleton, path tracking is performed along the skeleton from the proximal to the distal endpoint to obtain an ordered sequence of skeleton pixels. Since distal vein regions are often highly curved, fitting a line to the entire skeleton may introduce directional bias and weaken the representation of vein orientation near the stem region. To address this, only the proximal portion of the skeleton is used for local trend estimation. Specifically, the first 30% of the ordered skeleton points starting from the proximal endpoint is selected as the proximal leaf vein skeleton (**Figure 6C**). The selection of this proximal skeleton ratio is further evaluated in Section 3.5.1. The selected points are then fitted with a straight line to obtain a local leaf vein trend line. Before fitting, the points are mean-centered, and singular value decomposition (SVD) is applied to extract the principal orientation of the proximal leaf vein skeleton (**Figure 6C**). Each leaf vein is thus represented by one local trend line.

After multiple local leaf vein trend lines are obtained within the same canopy, pairwise intersections of these trend lines are calculated to construct a candidate stem point set (**Figure 6D**). For any two local leaf vein trend lines, if their direction vectors are not parallel or approximately parallel, the intersection point is obtained by solving the corresponding linear equations. These intersections are used as candidate stem points because the inward extensions of primary leaf veins tend to converge near the tobacco stem region. To remove invalid candidate stem points, two spatial constraints are introduced (**Figure 6D**). First, each candidate point must be located inside the corresponding canopy polygon. This constraint ensures that the predicted stem point remains within the biological region of the target plant. Second, each candidate point must be located within the minimum enclosing circle calculated from the proximal endpoints of the leaf veins assigned to the same canopy. This constraint further restricts candidate points to the central convergence region defined by the proximal endpoints.

After candidate stem points satisfying the spatial constraints are obtained, an iterative candidate-point filtering strategy is applied to improve localization stability (**Figure 6E**). When the number of candidate stem points is greater than three, the centroid of all current candidate points is calculated and defined as a virtual center. The candidate point farthest from this virtual center is then removed as a potential outlier. This process is repeated until only three candidate points remain. Finally, the mean coordinate of the remaining candidate points is used as the predicted stem point of the canopy (**Figure 6F**). Retaining three candidate points avoids relying on a single intersection while reducing the influence of dispersed outliers, thereby improving the robustness of the final prediction.

To ensure robustness, abnormal cases are handled as follows. If fewer than two valid leaf veins are assigned to a canopy, trend-line intersections cannot be computed, and no localization result is produced. If all candidate intersections fail to satisfy the canopy-region or minimum enclosing-circle constraints, the sample is regarded as a failure case. When valid candidate points are available, different strategies are applied depending on their number. If there are 1–3 candidates, their mean coordinate is directly used as the final stem prediction. If more than three candidates exist, an iterative virtual-center filtering strategy is applied to reduce the set to three points, and their mean coordinate is used as the final result.

#### 2.3.2 Evaluation metrics for stem point localization methods

To comprehensively evaluate the performance of the proposed stem point localization method, two aspects were considered: localization feasibility and localization accuracy.

First, the successful localization rate (*SLR*) was introduced to evaluate whether the proposed framework could successfully generate stem point predictions for tobacco plants. The *SLR* was calculated as follows:


$$SLR=\frac{N_{s}}{N_{t}}\times 100\%$$

where *N_s_* is the number of tobacco plants with successfully generated stem point predictions; *N_t_* is the total number of evaluated plants.

To evaluate the localization accuracy of the proposed method, the ground-truth stem point pixel coordinates $\left(x_{g,i},y_{g,i}\right)$ were manually annotated. The proposed method was then used to obtain the predicted stem point coordinates $\left(x_{p,i},y_{p,i}\right)$. For each plant, the Euclidean pixel distance error (*E_i_*) was calculated as follows:


$$E_{i}=\sqrt{{\left(x_{p,i}-x_{g,i}\right)}^{2}+{\left(y_{p,i}-y_{g,i}\right)}^{2}}\;\left(i=1,2\ldots ,N\right)$$

where *E_i_* is the Euclidean pixel distance error between the predicted stem point and the ground-truth stem point of the *i*-th tobacco plant; *N* is the number of tobacco plant samples.

The mean absolute error (*MAE*) was calculated as the average Euclidean localization error:


$$MAE=\frac{1}{N}\sum_{i=1}^{N}E_{i}$$

where *MAE* represents the average Euclidean pixel distance error between the predicted stem point and the ground-truth stem point.

The root mean square error (*RMSE*) was calculated as follows:


$$RMSE=\sqrt{\frac{1}{N}\sum_{i=1}^{N}\left[{\left(x_{p,i}-x_{g,i}\right)}^{2}+{\left(y_{p,i}-y_{g,i}\right)}^{2}\right]}$$

where *RMSE* is used to characterize the overall deviation between the predicted stem point coordinates and the ground-truth coordinates.

All localization errors were calculated in the original 1280 × 1280 image coordinate system. *RMSE* characterizes the overall localization deviation between the predicted stem point coordinates and the ground-truth coordinates. Compared with *MAE*, *RMSE* assigns greater weight to larger localization errors and is therefore more sensitive to outliers.

In addition, the median Euclidean pixel distance error (*E_med_*) and maximum Euclidean pixel distance error (*E_max_*) were calculated to describe the central tendency and worst-case deviation of the localization errors. The *E_med_* reflects the typical localization performance, whereas the *E_max_* indicates the largest localization deviation among all test samples. To further evaluate the practical robustness of different localization methods, the localization success rate under different pixel-error thresholds was calculated as follows:


$$SR\left(r\right)=\frac{1}{N}\sum_{i=1}^{N}I\left(E_{i}\leq r\right)\times 100\%$$

where *SR*(*r*) denotes the proportions of samples with the Euclidean pixel distance error not exceeding *r* px; *r* denotes the predefined pixel-error threshold, and *I*(·) is an indicator function defined as:


$$I\left(E_{i}\leq r\right)=\{\begin{array}{c} 1,\;E_{i}\leq r\\ 0,\;E_{i}>r \end{array}$$

In this study, *SR*(10), *SR*(20), and *SR*(30) were used to represent the proportions of samples with the Euclidean pixel distance error not exceeding 10 px, 20 px, and 30 px, respectively.

#### 2.3.3 Experimental design for error-propagation analysis

The proposed stem point localization framework consists of four sequential stages: canopy and leaf vein segmentation, leaf vein skeleton extraction, local direction fitting, and topology-guided stem point inference. Since the localization process relies on the structural information extracted from segmented leaf veins, segmentation errors may propagate through subsequent geometric processing and finally influence the localization accuracy. Therefore, segmentation performance and final localization accuracy alone cannot fully characterize the error sources of the complete localization pipeline.

To examine the downstream influence associated with different segmentation-mask sources, a controlled analysis was conducted on 100 images selected from the held-out test set, containing 213 tobacco plants. Three mask conditions were evaluated:

1. Ground-truth masks + proposed geometric inference: manually annotated canopy and leaf vein masks were used to estimate the residual error of the downstream geometric inference procedure.
2. YOLO-ODS masks + proposed geometric inference: predicted masks from YOLO-ODS were used to evaluate the complete proposed pipeline under practical conditions.
3. YOLOv11n-Seg masks + proposed geometric inference: predicted masks from the baseline segmentation model were used to analyze how reduced segmentation quality affects downstream localization.
  Except for the source of segmentation masks, all subsequent procedures, including skeleton extraction, proximal vein selection, local direction fitting, candidate stem point generation, spatial constraint filtering, and candidate-point optimization, were identical.

For each mask condition, all 213 tobacco plants were first evaluated to calculate the successful localization rate (*SLR*). Localization failures occurred when insufficient valid leaf veins were available, valid trend-line intersections could not be generated, or all candidate points were removed by spatial constraints. To evaluate localization accuracy after successful prediction, only tobacco plants successfully localized under all three mask conditions were retained. A total of 190 commonly localized plants were selected, and *MAE*, *RMSE*, *E_max_*, *E_med_*, *SR*(10), *SR*(20), and *SR*(30) were calculated.

The ground-truth-mask condition was used as an empirical reference for the residual error of the downstream geometric pipeline rather than as a strict theoretical lower bound. The differences between the ground-truth-mask condition and the predicted-mask conditions were interpreted as the additional downstream performance degradation associated with replacing manually annotated masks with model-predicted masks. Because canopy and leaf vein masks were changed jointly, the present experiment quantified their combined downstream influence and did not separately estimate the independent contributions of canopy-mask and leaf vein mask errors.

#### 2.3.4 Comparison of stem point localization methods

To comprehensively evaluate the effectiveness of the proposed topology-guided stem point localization method, three representative baseline localization strategies were selected for comparison under identical datasets and experimental conditions: a keypoint-regression-based method, a bounding-box-center-based method, and a canopy-centroid-based method.

The first comparison method was a keypoint-regression-based localization strategy implemented using YOLOv11n-pose (Chen et al., 2024). In this approach, the stem point of each tobacco plant was manually annotated as a keypoint, and a YOLOv11n-pose model was trained using the same data split and experimental environment. The optimal model weights obtained during training were then used to predict stem point coordinates in the test images. This method represents a learning-based localization strategy that directly learns the mapping from image appearance features to stem point coordinates.

The second comparison method was a bounding-box-center-based localization strategy. Specifically, the canopy detection results generated by YOLO-ODS were used to obtain the bounding box of each tobacco canopy, and the geometric center of the bounding box was directly regarded as the predicted stem point. This method represents a simple geometric localization strategy based on canopy detection results (Sun et al., 2020).

The third comparison method was a canopy-centroid-based localization strategy. For each canopy instance segmented by YOLO-ODS, the geometric centroid of the canopy mask region was calculated and used as the predicted stem point coordinate. This method represents a geometric localization strategy based on canopy segmentation results and is commonly used when explicit stem point detection or keypoint annotation is unavailable (Zhang et al., 2022; Li et al., 2025). The proposed topology-guided localization method and the three comparison methods were evaluated using the same low-altitude nadir-view UAV images. For a fair comparison, all methods used the same manually annotated stem point ground-truth coordinates and identical test samples.

For fair comparison, all four localization methods were evaluated using identical UAV images and manually annotated stem point coordinates. A total of 70 tobacco plants were selected from low-altitude nadir-view UAV images collected after topping at a flight height of 6 m. The upper stem regions of these plants were visible and manually identifiable, allowing accurate annotation of ground-truth stem points. All four methods were first applied to these 70 plants, and the *SLR* was calculated. Subsequently, only plants successfully localized by all four methods were retained for localization accuracy evaluation. According to the criterion that the visible upper stem region could be reliably annotated, 50 commonly localized plants were selected. The localization performance of the four methods was quantified using *MAE*, *RMSE*, *E_max_*, *E_med_*, *SR*(10), *SR*(20), and *SR*(30).

To provide a more intuitive evaluation of the stem point localization accuracy, the pixel-level localization errors were converted into physical distances based on the ground sampling distance (*GSD*) of the UAV images. *GSD* represents the ground distance corresponding to one image pixel and was calculated according to the UAV flight altitude and camera parameters. The *GSD* under nadir-view imaging and approximately flat terrain conditions was calculated as follows:


$$GSD=\frac{H\times S_{w}}{f\times I_{w}}$$

where *H* represents the flight altitude, *S*_w_ is the sensor width, *f* represents the actual focal length, and *I*_w_ represents the image width in pixels.

The UAV platform used in this study was a DJI Mavic 3 equipped with a 4/3 CMOS Hasselblad camera. According to the camera specifications, the sensor width and actual focal length were approximately 17.4 mm and 12.29 mm, respectively. The original UAV images had a resolution of 5280 × 3956 pixels. For the stem point localization experiment, images were collected at a flight height of approximately 6 m. Therefore, using *H* = 6000, *S*_w_ =17.4 mm, *f* = 12.29 mm, and *I*_w_ =5280 pixels, the calculated *GSD* was approximately 1.61 mm/pixel.

### 2.4 Evaluation metrics for instance segmentation models

To clarify the evaluation protocol, all segmentation accuracy metrics reported in this study were calculated based on instance masks rather than bounding boxes. The evaluated metrics included mask Precision, mask Recall, mask F1-score, mask mAP@0.5, and mask mAP@0.5:0.95. In addition, model size, parameter count, and GFLOPs were used to evaluate computational complexity and deployment efficiency. During evaluation on the test set, the trained models were used to generate instance mask predictions using the Ultralytics YOLO instance segmentation validation procedure. A minimum confidence threshold of 0.25 was used during validation and post-processing to exclude extremely low-confidence predictions, and an NMS IoU threshold of 0.50 was adopted to suppress redundant overlapping predictions. These inference and post-processing parameters were not involved in model training or parameter optimization.

The segmentation metrics were calculated using the instance segmentation evaluation implementation of Ultralytics YOLO. Predicted instance masks were matched with ground-truth masks according to their mask IoU values. A mask IoU threshold of 0.50 was adopted as the matching criterion for determining true positives (*TP*), false positives (*FP*), and false negatives (*FN*). Correctly matched predicted masks were regarded as *TP*, unmatched predicted masks were regarded as *FP*, and unmatched ground-truth masks were regarded as *FN*. For each category, mask Precision, mask Recall, and mask F1-score were determined at the confidence operating point corresponding to the maximum mean mask F1-score and were calculated as follows:


$$mask\;Precision=\frac{TP}{TP+FP}\times 100\%$$

$$mask\;Recall=\frac{TP}{TP+FN}\times 100\%$$

$$mask\;F1-score=\frac{2\times mask\;Precision\times mask\;Recall}{mask\;Precision+\text{mask Recall}}\times 100\%$$

The mask AP for each category was calculated as the area under the corresponding mask Precision-Recall curve. Mask mAP@0.5 was calculated by averaging category-specific mask AP values at a mask IoU threshold of 0.50. Mask mAP@0.5:0.95 was obtained by averaging AP values across IoU thresholds ranging from 0.50 to 0.95 with an interval of 0.05.

Two instance segmentation categories, canopy and leaf vein, were evaluated in this study. The overall mask Precision and mask Recall were calculated using unweighted macro-averaging, namely, by taking the arithmetic mean of the corresponding category-specific values. The overall mask F1-score was then calculated from the resulting overall mask Precision and mask Recall using the standard F1-score formula. The overall mask mAP@0.5 and mask mAP@0.5:0.95 were also calculated using unweighted arithmetic averaging across the two categories. No weighting according to the number of instances in each category was applied. Category-specific mask AP@0.5 and mask AP@0.5:0.95 were additionally reported to evaluate segmentation performance for canopy and leaf vein separately.

## 3 Results and analysis

### 3.1 Experimental setup

All experiments were conducted on a Windows 11 operating system. The hardware configuration included an NVIDIA RTX 4090 GPU, an Intel Core i9-14900K CPU. The software environment included Python 3.10, PyTorch 2.2.2, CUDA 12.1, and Ultralytics 8.4.52.

To ensure fair comparison among models, the same training settings were used in all experiments. The input image size was set to 640 × 640, the batch size was set to 16, and the number of training epochs was set to 200. The AdamW optimizer was used during training, with an initial learning rate of 0.007. Other hyperparameters followed the default configuration of the YOLOv11n-Seg baseline model. Furthermore, to investigate the effect of input image resolution on model performance, additional validation experiments with different input sizes were conducted. Detailed descriptions of the experimental settings and results are provided in **Supplementary Material** Section 3, as shown in **Supplementary Tables S5** and **S6**. An input size of 640 × 640 was selected as a compromise between retaining sufficient fine-scale leaf vein information and reducing computational cost for UAV-based agricultural applications.

### 3.2 Ablation study of YOLO-ODS components

To evaluate the effectiveness of the main components of YOLO-ODS, YOLOv11n-Seg was used as the baseline model. OACM, DIAM, and Slide Loss were progressively introduced into the baseline framework, and their effects on tobacco canopy and leaf vein instance segmentation were evaluated under the same data split, training settings, and experimental environment. The experimental results are shown in **Tables 3** and **4**.

**Table 3:** Ablation results of YOLO-ODS components on overall instance segmentation performance.

Model	Mask precision	Mask recall	Mask F1-score	Mask mAP@0.5	Mask mAP@0.5:0.95	Model size (MB)	Parameters (M)	GFLOPs
YOLOv11n-Seg	85.55%	84.59%	85.07%	89.05%	65.10%	5.75	2.84	9.73
YOLOv11n-Seg+OACM	86.72%	85.24%	85.97%	89.97%	66.02%	5.79	2.85	10.19
YOLOv11n-Seg+DIAM	87.24%	83.55%	85.36%	89.76%	66.03%	5.78	2.85	9.73
YOLOv11n-Seg+Slide Loss	84.77%	85.82%	85.29%	90.59%	66.51%	5.75	2.84	9.73
YOLOv11n-Seg+OACM+Slide Loss	86.08%	84.75%	85.41%	89.31%	66.03%	5.79	2.85	10.19
YOLOv11n-Seg+DIAM+Slide Loss	86.66%	84.48%	85.56%	89.85%	66.55%	5.77	2.85	9.73
YOLOv11n-Seg+OACM+DIAM	85.79%	86.26%	86.02%	90.12%	66.90%	5.81	2.86	10.19
YOLO-ODS(YOLOv11n-Seg+OACM+DIAM+Slide Loss)	85.69%	86.79%	86.24%	90.19%	67.20%	5.81	2.86	10.19

**Table 4:** Ablation results of YOLO-ODS components on category-level instance segmentation performance.

Model	Canopy		Leaf vein	
	Mask mAP@0.5	Mask mAP@0.5:0.95	Mask mAP@0.5	Mask mAP@0.5:0.95
YOLOv11n-Seg	93.46%	81.11%	84.63%	49.08%
YOLOv11n-Seg+OACM	94.42%	81.63%	85.52%	50.41%
YOLOv11n-Seg+DIAM	94.40%	81.98%	85.11%	50.08%
YOLOv11n-Seg+Slide Loss	94.45%	81.91%	86.74%	51.11%
YOLOv11n-Seg+OACM+Slide Loss	92.75%	81.18%	85.86%	50.88%
YOLOv11n-Seg+DIAM+Slide Loss	94.24%	82.83%	85.45%	50.27%
YOLOv11n-Seg+OACM+DIAM	94.96%	82.95%	85.28%	50.85%
YOLO-ODS(YOLOv11n-Seg+OACM+DIAM+Slide Loss)	94.60%	82.91%	85.78%	51.49%

As shown in **Table 3**, the baseline YOLOv11n-Seg achieved a mask Precision of 85.55%, mask Recall of 84.59%, mask F1-score of 85.07%, mask mAP@0.5 of 89.05%, and mask mAP@0.5:0.95 of 65.10%. After introducing OACM alone, the mask mAP@0.5:0.95 increased to 66.02%, indicating that directional feature enhancement improves the representation capability of slender and structurally continuous targets. DIAM alone improved the mask mAP@0.5:0.95 to 66.03%, demonstrating that attention-based feature recalibration can effectively enhance feature discrimination under complex field backgrounds. Compared with the two structural modules, Slide Loss alone achieved a more significant improvement in overall segmentation accuracy. It increased mask mAP@0.5 from 89.05% to 90.59% and mask mAP@0.5:0.95 from 65.10% to 66.51%. At the category level, Slide Loss improved the leaf vein mask mAP@0.5 and mask mAP@0.5:0.95 from 84.63% and 49.08% to 86.74% and 51.11%, respectively. These results indicate that the dynamic loss weighting strategy effectively improves the optimization of difficult samples, particularly for slender and weak-texture leaf vein structures.

The combination experiments further showed that the effects of the three components were not simply additive. Among all two-component configurations, OACM+DIAM achieved the best overall performance, with a mask Recall of 86.26%, mask F1-score of 86.02%, mask mAP@0.5 of 90.12%, and mask mAP@0.5:0.95 of 66.90%. The favorable performance of this combination suggests a complementary relationship between orientation-sensitive feature extraction and attention-based feature recalibration. OACM strengthens responses to directional structures, whereas DIAM selectively recalibrates informative spatial regions and semantic channels, thereby allowing useful directional features to be retained while suppressing less relevant responses.

In contrast, OACM+Slide Loss did not provide an additional overall improvement. OACM alone increased mask mAP@0.5:0.95 from 65.10% to 66.02%, and Slide Loss alone increased it to 66.51%, whereas their combination achieved 66.03%. The category-level results in **Table 4** provide further insight into this non-additive behavior. OACM+Slide Loss achieved a leaf vein mask mAP@0.5:0.95 of 50.88%, which remained 1.80 percentage points higher than the baseline value of 49.08%. However, its canopy mask mAP@0.5 decreased to 92.75%, compared with 94.42% for OACM alone and 94.45% for Slide Loss alone. Therefore, the absence of an additional overall gain was not caused by a uniform deterioration in both categories. Instead, the improvement retained for fine-grained leaf vein segmentation was partly offset by reduced canopy performance, resulting in a redistribution of performance between the two target categories. This non-additive behavior may be associated with the different but coupled functional levels of OACM and Slide Loss. OACM primarily modifies feature representation by strengthening orientation-sensitive responses associated with leaf veins, leaf boundaries, and other elongated structures. Changes in feature representation can subsequently alter prediction confidence, mask quality, and the IoU-based matching-quality distribution of training samples. Slide Loss, in contrast, dynamically adjusts the optimization contribution of samples according to their matching qualities. Therefore, once OACM modifies the underlying feature representation, the distribution of matching qualities encountered by Slide Loss may also change, which in turn changes the relative weighting of different training samples. Consequently, the effect of Slide Loss after OACM is introduced is not necessarily equivalent to its effect on the original baseline representation. This coupling between feature representation and sample-quality-aware optimization provides a plausible explanation for why the individual gains of OACM and Slide Loss were not fully accumulated when they were directly combined.

The DIAM+Slide Loss configuration showed a different but similarly non-additive interaction. Its mask mAP@0.5:0.95 reached 66.55%, which was only 0.04 percentage points higher than that of Slide Loss alone. Thus, the present results do not indicate a strong synergistic effect between these two components when used independently of OACM. Nevertheless, because DIAM performs spatial-channel feature recalibration before loss-based optimization, it may provide a more discriminative representation for subsequent sample-quality weighting.

When all three components were integrated, the proposed YOLO-ODS achieved the best overall segmentation performance, with a mask Recall of 86.79%, mask F1-score of 86.24%, mask mAP@0.5 of 90.19%, and mask mAP@0.5:0.95 of 67.20%. Compared with the baseline YOLOv11n-Seg, YOLO-ODS improved mask Recall, mask F1-score, mask mAP@0.5, and mask mAP@0.5:0.95 by 2.20, 1.17, 1.14, and 2.10 percentage points, respectively. Meanwhile, the number of parameters and GFLOPs remained almost unchanged, demonstrating that the proposed improvements enhance segmentation performance without significantly increasing computational complexity.

The category-level results in **Table 4** further demonstrate the effectiveness of the proposed components. For canopy segmentation, OACM+DIAM achieved the highest mask mAP@0.5 and mask mAP@0.5:0.95 values of 94.96% and 82.95%, respectively, indicating that the combination of directional feature modeling and attention refinement is beneficial for large-scale canopy structures. After introducing Slide Loss, the canopy performance remained comparable, with only slight changes in accuracy. For leaf vein segmentation, Slide Loss alone achieved a high mask mAP@0.5 of 86.74%, demonstrating its ability to improve the detection of difficult vein instances. However, the complete YOLO-ODS model achieved the highest leaf vein mask mAP@0.5:0.95 of 51.49%, improving by 2.41 percentage points compared with the baseline model. Compared with the OACM+DIAM configuration, adding Slide Loss further increased leaf vein mask mAP@0.5 and mask mAP@0.5:0.95 by 0.50 and 0.64 percentage points, respectively. This improvement is particularly important because accurate leaf vein segmentation provides the structural basis for subsequent topology-guided stem point localization.

Overall, the complete ablation experiments demonstrate that OACM, DIAM, and Slide Loss contribute to YOLO-ODS from different perspectives. OACM improves directional feature representation, DIAM enhances spatial and semantic feature discrimination, and Slide Loss optimizes difficult sample learning. Their combination does not produce a simple additive effect; instead, the interaction among modules determines the final performance. The complete YOLO-ODS framework achieves the best balance between segmentation accuracy, fine-grained leaf vein representation, and computational efficiency.

### 3.3 Comparison of different attention mechanisms

To further evaluate the effectiveness of the proposed Dual Interactive Attention Module (DIAM), additional comparative experiments were conducted using several representative attention mechanisms, including Squeeze-and-Excitation (SE), Efficient Channel Attention (ECA), Convolutional Block Attention Module (CBAM), and Coordinate Attention (CA). The YOLOv11n-Seg+OACM+Slide Loss configuration without an additional attention module was included as the reference configuration. For a fair comparison, all attention modules were inserted at the same network position, while the backbone architecture, OACM, Slide Loss, dataset partition, and training strategy were kept unchanged. All experiments were conducted using the experimental environment and parameter settings described in Section 3.1. The overall and category-level comparison results are presented in **Tables 5** and **6**, respectively.

**Table 5:** Comparison of different attention mechanisms in terms of overall instance segmentation performance.

Model	Mask precision	Mask recall	Mask F1-score	Mask mAP@0.5	Mask mAP@0.5:0.95	Model size (MB)	Parameters (M)	GFLOPs
YOLOv11n-Seg+OACM+Slide Loss	86.08%	84.75%	85.41%	89.31%	66.03%	5.79	2.85	10.19
YOLOv11n-Seg+OACM+CBAM+Slide Loss	84.18%	85.46%	84.82%	88.92%	65.69%	5.81	2.86	10.19
YOLOv11n-Seg+OACM+SE+Slide Loss	86.19%	84.13%	85.14%	89.51%	65.69%	5.79	2.85	10.19
YOLOv11n-Seg+OACM+ECA+Slide Loss	84.58%	86.19%	85.38%	89.83%	66.61%	5.79	2.85	10.19
YOLOv11n-Seg+OACM+CA+Slide Loss	85.10%	84.68%	84.89%	89.72%	66.36%	5.80	2.86	10.19
YOLO-ODS (YOLOv11n-Seg+OACM+DIAM+Slide Loss)	85.69%	86.79%	86.24%	90.19%	67.20%	5.81	2.86	10.19

**Table 6:** Comparison of different attention mechanisms on category-level instance segmentation performance.

Model	Canopy		Leaf vein	
	Mask mAP@0.5	Mask mAP@0.5:0.95	Mask mAP@0.5	Mask mAP@0.5:0.95
YOLOv11n-Seg+OACM+Slide Loss	92.75%	81.18%	85.86%	50.88%
YOLOv11n-Seg+OACM+CBAM+Slide Loss	93.06%	81.18%	84.79%	50.21%
YOLOv11n-Seg+OACM+SE+Slide Loss	93.58%	80.69%	85.43%	50.69%
YOLOv11n-Seg+OACM+ECA+Slide Loss	94.31%	82.85%	85.35%	50.37%
YOLOv11n-Seg+OACM+CA+Slide Loss	93.93%	81.79%	85.51%	50.90%
YOLO-ODS (YOLOv11n-Seg+OACM+DIAM+Slide Loss)	94.60%	82.91%	85.78%	51.49%

As shown in **Table 5**, the introduction of different attention mechanisms produced distinct performance changes relative to the YOLOv11n-Seg+OACM+Slide Loss reference configuration. The results indicate that the effectiveness of an attention mechanism was dependent on its feature-recalibration strategy, and not all attention modules produced consistent improvements across all evaluation metrics. CBAM and SE did not improve the overall mask mAP@0.5:0.95 relative to the reference configuration, whereas ECA, CA, and DIAM achieved numerical gains of 0.58, 0.33, and 1.17 percentage points, respectively. Among all evaluated configurations, YOLO-ODS equipped with DIAM achieved the highest mask Recall, mask F1-score, mask mAP@0.5, and mask mAP@0.5:0.95, reaching 86.79%, 86.24%, 90.19%, and 67.20%, respectively. Compared with the configuration without an additional attention module, DIAM increased mask Recall by 2.04 percentage points, mask F1-score by 0.83 percentage points, mask mAP@0.5 by 0.88 percentage points, and mask mAP@0.5:0.95 by 1.17 percentage points. Although mask Precision decreased slightly from 86.08% to 85.69%, the substantial increase in mask Recall resulted in a more favorable balance between false-positive suppression and instance recovery.

Compared with CBAM, SE, ECA, and CA, DIAM improved overall mask mAP@0.5:0.95 by 1.51, 1.51, 0.59, and 0.84 percentage points, respectively. It also achieved the highest overall mask mAP@0.5, exceeding the second-best ECA configuration by 0.36 percentage points. Meanwhile, the parameter counts and computational costs of all attention-enhanced models remained similar, ranging from 2.85 M to 2.86 M parameters and remaining at approximately 10.19 GFLOPs. Therefore, the improved performance of DIAM was not associated with a substantial increase in model complexity.

The category-level results in **Table 6** provide further insight into the performance differences among the attention mechanisms. For canopy segmentation, DIAM achieved mask mAP@0.5 and mask mAP@0.5:0.95 values of 94.60% and 82.91%, respectively, representing improvements of 1.85 and 1.73 percentage points over the reference configuration without an additional attention module. DIAM also outperformed the second-best ECA configuration by 0.29 percentage points in canopy mask mAP@0.5 and by 0.06 percentage points in canopy mask mAP@0.5:0.95. These results suggest that the joint modeling of spatial and channel information improved the discrimination and delineation of canopy instances under complex field conditions. For leaf vein segmentation, the reference configuration achieved the highest mask mAP@0.5 of 85.86%, whereas DIAM obtained a comparable value of 85.78%, corresponding to a slight decrease of 0.08 percentage points. However, DIAM increased leaf vein mask mAP@0.5:0.95 from 50.88% to 51.49%, representing an improvement of 0.61 percentage points. It also outperformed the second-best attention mechanism, CA, by 0.59 percentage points in this stricter metric. The nearly unchanged mask mAP@0.5, together with the improvement in mask mAP@0.5:0.95, suggests that DIAM primarily enhanced leaf vein mask quality under more stringent overlap requirements rather than merely increasing the number of instances detected at an IoU threshold of 0.50. This characteristic is particularly relevant to subsequent skeleton extraction and local direction fitting, which depend on the continuity and geometric accuracy of predicted leaf vein masks.

The different behaviors of SE, ECA, CBAM, CA, and DIAM can be further interpreted according to their feature-recalibration mechanisms and the structural characteristics of the present task. Tobacco canopy and primary leaf vein segmentation involves two substantially different types of targets. Canopy instances occupy relatively large and spatially continuous regions, whereas primary leaf veins are slender, low-contrast, spatially sparse, and distributed over multiple orientations. In the constructed dataset, leaf vein pixels account for only approximately 1.40% of the total image area, compared with approximately 35.73% for canopy pixels. Consequently, an attention mechanism suitable for this task should preserve weak local responses from fine-scale veins while simultaneously maintaining sufficient semantic discrimination for large canopy regions.

SE performs feature recalibration exclusively along the channel dimension using globally aggregated descriptors. This strategy is effective for identifying globally important semantic channels but does not explicitly encode the spatial locations of fine-scale structures. For the present task, the responses generated by slender primary leaf veins occupy only a small proportion of the feature map. Global spatial aggregation may therefore provide limited information about where these weak and spatially sparse responses occur. This may partly explain why SE did not improve the overall mask mAP@0.5:0.95 under the present network configuration.

ECA also focuses primarily on channel-wise recalibration but introduces efficient local cross-channel interaction without dimensionality reduction. Its relatively strong overall performance indicates that preserving local dependencies among neighboring channels is beneficial for the present segmentation task. However, because ECA does not explicitly generate a spatial attention map, its ability to distinguish weak leaf vein regions from surrounding leaf tissues, shadows, soil, and other spatially localized interference remains dependent on the preceding convolutional features. This may limit its ability to simultaneously optimize large canopy regions and spatially sparse fine-grained leaf vein structures.

CBAM sequentially applies channel attention followed by spatial attention and therefore incorporates both types of information. Nevertheless, under the present network configuration, CBAM did not improve the overall mask mAP metrics relative to the reference model. One possible explanation is that sequential attention makes the spatial recalibration stage dependent on features that have already undergone channel-wise modulation. For weak and sparse structures such as primary leaf veins, an unfavorable channel response at the first stage could reduce the information subsequently available to the spatial branch. However, this interpretation remains a possible explanation rather than direct evidence that CBAM causes information loss, and its observed performance should be regarded as task- and configuration-dependent.

Coordinate Attention introduces positional information by separately aggregating features along the horizontal and vertical spatial directions. This design provides stronger spatial awareness than channel-only mechanisms and resulted in improved overall performance relative to the reference configuration. However, tobacco primary leaf veins generally extend radially from the stem region and may appear in horizontal, vertical, diagonal, or curved orientations depending on plant morphology and UAV viewing geometry. Therefore, spatial encoding based primarily on two orthogonal coordinate directions may not fully correspond to the multi-directional structural distribution of leaf veins. This may explain why CA provided a measurable improvement but remained below DIAM in overall and strict leaf vein mask metrics.

In contrast, DIAM generates spatial and channel attention representations in parallel rather than relying exclusively on channel information or on a sequential channel-to-spatial recalibration process. The spatial branch identifies informative regions directly from the input feature representation, which can help preserve weak local responses associated with slender leaf veins and canopy boundaries. In parallel, the channel branch emphasizes semantic channels associated with tobacco structural information. The resulting spatial and channel attention are jointly applied to the original feature representation, allowing spatial localization and semantic discrimination to contribute simultaneously to feature recalibration.

This parallel design is particularly compatible with the heterogeneous structural characteristics of the present segmentation task. For large canopy instances, channel-level semantic recalibration helps distinguish tobacco plants from soil, weeds, shadows, and neighboring vegetation, while spatial attention supports instance boundaries and overlapping canopy regions. For primary leaf veins, the spatial branch can emphasize locally distributed fine structures, while the channel branch retains feature channels responsive to vein appearance and structural information. Thus, DIAM does not rely on a single type of attention to address both categories but combines complementary spatial and semantic cues within the same recalibration stage.

The interaction between DIAM and OACM may also contribute to the observed performance. OACM explicitly enhances horizontal, vertical, and diagonal structural responses before attention-based recalibration. DIAM can subsequently use spatial and channel information to selectively emphasize task-relevant orientation-enhanced responses while suppressing responses from other directional patterns such as leaf edges, shadows, or background textures. This provides a plausible functional complementarity between orientation-sensitive feature extraction and parallel spatial-channel attention and is consistent with the strong performance observed for the OACM+DIAM combination in the component ablation experiments.

Overall, the comparative experiments indicate that the advantage of DIAM in the present task is associated not merely with introducing additional attention operations, but with the compatibility between its parallel spatial-channel feature recalibration and the coexistence of large canopy regions and sparse, slender, multi-directional primary leaf veins. In particular, the improvement in leaf vein mask mAP@0.5:0.95 without a corresponding increase in mask mAP@0.5 suggests that DIAM mainly contributes to more accurate fine-grained mask representation under strict overlap requirements. Nevertheless, these results demonstrate the relative suitability of DIAM under the current dataset, network position, and training configuration and should not be interpreted as evidence that DIAM is universally superior to SE, ECA, CBAM, or CA across other tasks or architectures.

### 3.4 Performance and deployment analysis of YOLOv11n-Seg and YOLO-ODS

To comprehensively evaluate the segmentation performance and deployment feasibility of YOLO-ODS, category-specific mask Precision-Recall curves, normalized category-level confusion matrices, qualitative segmentation results, and embedded inference performance were analyzed on the test set. Representative images containing complex field conditions were also selected to compare the segmentation performance of YOLO-ODS and YOLOv11n-Seg.

The category-specific mask Precision-Recall curves were generated using the Ultralytics instance segmentation validation procedure with a minimum confidence threshold of 0.001. This low threshold retained predictions over a broad confidence range, while the confidence operating point was progressively varied to calculate mask Precision and mask Recall. Predicted and ground-truth instance masks belonging to the same category were matched using a mask IoU threshold of 0.50. The resulting curves therefore characterize the trade-off between mask Precision and mask Recall across different confidence operating points.

As shown in **Figure 7A**, the canopy mask Precision-Recall curves were generally higher than the leaf vein curves for both models, reflecting the greater spatial extent and more distinct morphological characteristics of canopy instances. YOLO-ODS exhibited a generally more favorable mask Precision-Recall profile than YOLOv11n-Seg for both categories, with the curve of YOLO-ODS consistently above that of the baseline over most of the evaluated recall range. For the canopy category, both models maintained relatively high mask Precision over a broad recall range. Nevertheless, YOLO-ODS retained higher Precision at relatively high Recall levels, indicating improved canopy-instance recovery without a corresponding substantial increase in false-positive predictions. Leaf vein segmentation remained more challenging because primary veins are slender, low in contrast, and occupy only a small proportion of image pixels. They are also susceptible to occlusion and visual confusion with leaf edges and surrounding textures. Although the leaf vein mask Precision-Recall curves were lower than the canopy curves, YOLO-ODS generally exhibited a more favorable Precision-Recall relationship than YOLOv11n-Seg.

**Figure 7:**
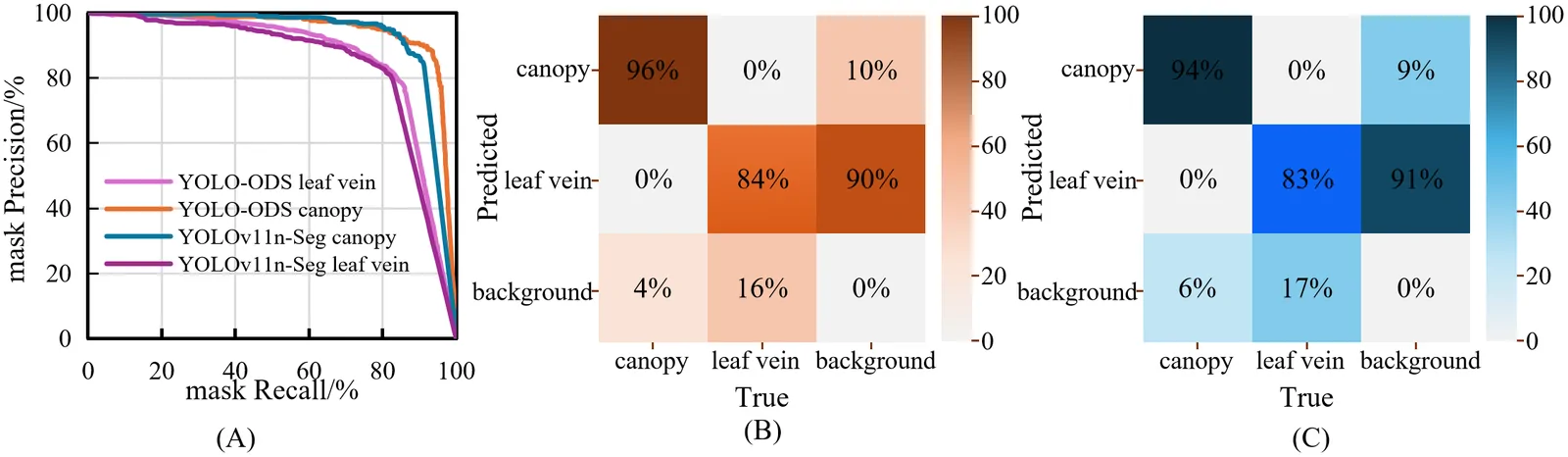
Mask Precision-Recall curves and category-level confusion matrices of YOLO-ODS and YOLOv11n-Seg on the test set. **(A)** Category-specific mask Precision-Recall curves of YOLO-ODS and YOLOv11n-Seg; **(B)** normalized category-level confusion matrix of YOLO-ODS; and **(C)** normalized category-level confusion matrix of YOLOv11n-Seg.

The normalized category-level confusion matrices were generated in a separate validation run using a fixed confidence threshold of 0.25 and an NMS IoU threshold of 0.50. After confidence filtering and non-maximum suppression, predicted instances were matched one-to-one with ground-truth instances according to the IoU of their associated bounding boxes. In the adopted Ultralytics implementation, a default box-IoU matching threshold of 0.45 was used for confusion-matrix accumulation. As shown in **Figures 7B**, YOLO-ODS correctly matched and classified 96% of the ground-truth canopy instances and 84% of the ground-truth leaf vein instances to their corresponding categories. The corresponding values for YOLOv11n-Seg were 94% and 83%, respectively, as shown in **Figure 7C**. Neither model exhibited observable intercategory confusion between canopy and leaf vein instances under the adopted normalization scheme. The proportion of ground-truth canopy instances assigned to the background decreased from 6% for YOLOv11n-Seg to 4% for YOLO-ODS, while the corresponding proportion for leaf vein instances decreased from 17% to 16%. These results indicate that YOLO-ODS achieved a modest but consistent reduction in missed instances, particularly for canopy segmentation.

Qualitative segmentation results are further presented in **Figure 8**. Under challenging field conditions, including dense planting, canopy overlap, weak leaf vein texture, shadows, and background interference, YOLOv11n-Seg exhibited incomplete canopy boundaries, missed or fragmented leaf vein instances, and inaccurate mask localization. In comparison, YOLO-ODS produced more complete canopy masks and improved the continuity and localization of primary leaf vein structures.

**Figure 8:**
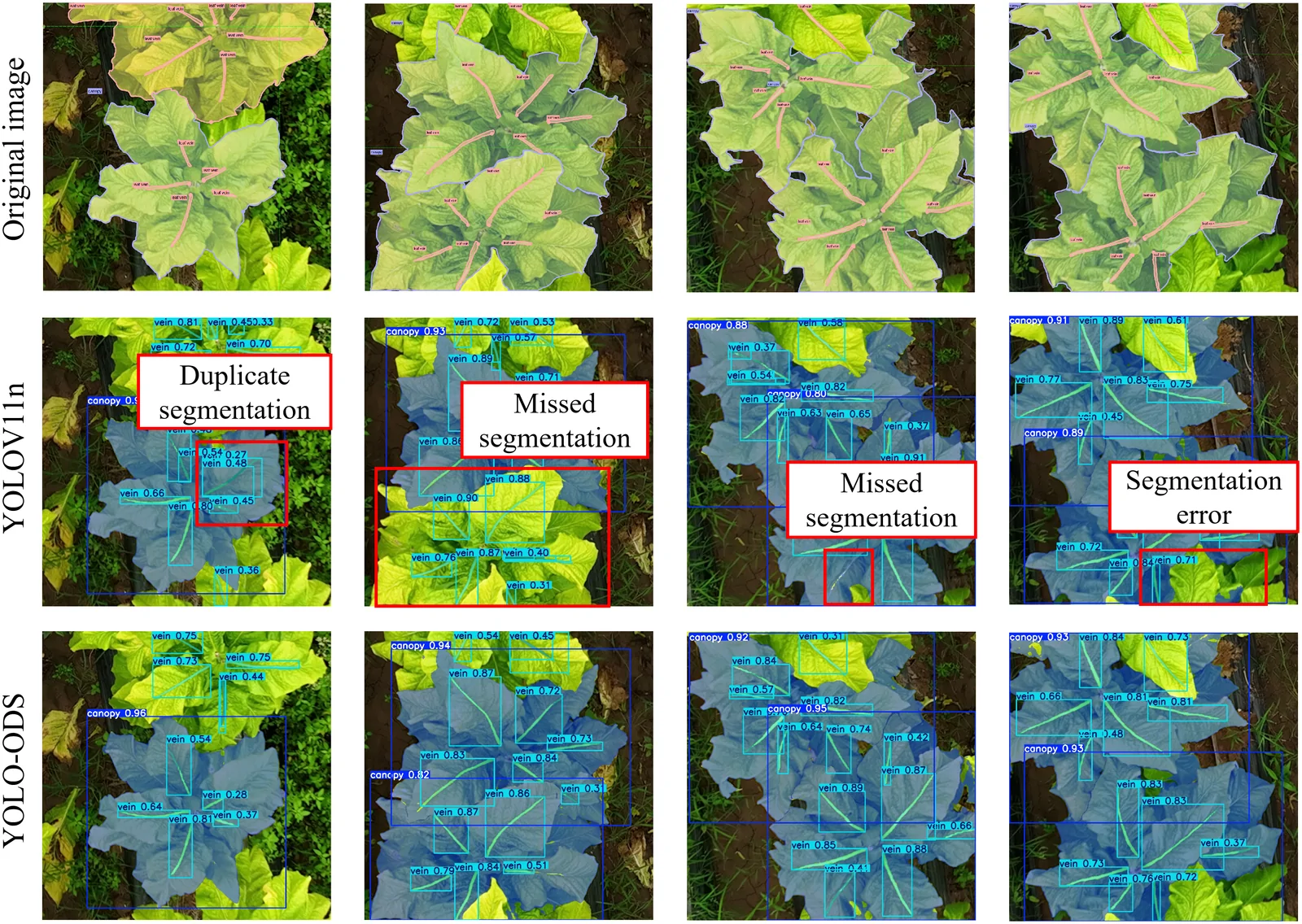
Visual comparison of tobacco canopy and leaf vein segmentation results between YOLOv11n-Seg and YOLO-ODS.

In addition to segmentation accuracy, the practical deployment efficiency of YOLO-ODS was further evaluated on an embedded edge computing platform. Although model parameters and GFLOPs provide useful information regarding theoretical computational complexity, they cannot fully represent actual inference efficiency under hardware constraints. Therefore, embedded inference experiments were conducted on an NVIDIA Jetson Orin Nano 8GB developer kit to evaluate the real-world deployment performance of the proposed model. The Jetson Orin Nano 8GB platform was configured with the maximum performance power mode (MAXN) and deployed using the TensorRT inference framework with FP16 precision acceleration. Both YOLOv11n-Seg and YOLO-ODS were converted into TensorRT engines under identical deployment settings. The input resolution was fixed at 640 × 640 pixels, and the batch size was set to 1 to simulate the inference scenario of UAV-based agricultural applications. The inference speed evaluation was conducted on the complete test set containing 261 UAV image patches. Before measurement, 50 warm-up iterations were performed to eliminate initialization overhead, and the average inference latency was calculated over 500 inference runs. The reported inference time included image preprocessing, network inference, and post-processing operations.

YOLO-ODS achieved an average inference latency of 43.2 ms and an inference speed of 24.7 FPS on the Jetson Orin Nano 8GB platform. Under the same conditions, YOLOv11n-Seg achieved an average inference latency of 38.6 ms and an inference speed of 26.9 FPS. Compared with YOLOv11n-Seg, YOLO-ODS introduced only a slight reduction in inference efficiency while providing improved segmentation performance. Specifically, YOLO-ODS increased the parameter number from 2.84 M to 2.86 M and GFLOPs from 9.73 to 10.19, representing only a minor increase in computational complexity.

These results demonstrate that the proposed modules improve segmentation accuracy while maintaining efficient inference capability on embedded hardware. Therefore, YOLO-ODS achieves a favorable balance between segmentation performance and deployment efficiency, indicating its potential for UAV-based tobacco phenotyping and intelligent agricultural operations.

### 3.5 Comparison with different instance segmentation models

To further verify the effectiveness of the proposed YOLO-ODS model, several mainstream instance segmentation models were selected for comparison, including YOLOv5n-Seg, YOLOv6n-Seg, YOLOv8n-Seg, YOLOv10n-Seg, YOLOv11n-Seg, YOLOv12n-Seg, YOLOv26n-Seg, and RT-DETR-ResNet50. The experimental results are shown in **Tables 7** and **8**.

**Table 7:** Comparison with representative instance segmentation models on overall instance segmentation performance.

Model	Mask precision	Mask recall	Mask F1-score	Mask mAP@0.5	Mask mAP@0.5:0.95	Model size (MB)	Parameters (M)	GFLOPs
YOLOv5n-Seg	85.77%	83.61%	84.68%	88.10%	64.64%	5.56	2.76	10.46
YOLOv6n-Seg	83.78%	85.36%	84.56%	88.95%	64.98%	8.66	4.41	14.63
YOLOv8n-Seg	85.59%	82.79%	84.17%	88.38%	64.88%	6.5	3.26	11.48
YOLOv10n-Seg	83.93%	85.20%	84.56%	88.76%	64.64%	5.15	2.53	9.95
YOLOv11n-Seg	85.55%	84.59%	85.07%	89.05%	65.10%	5.75	2.84	9.73
YOLOv12n-Seg	86.10%	83.05%	84.55%	88.94%	65.22%	5.8	2.82	9.77
YOLOv26n-Seg	85.26%	84.84%	85.05%	88.83%	65.37%	6.27	3.05	10.16
RT-DETR-ResNet50-Seg	82.88%	82.94%	82.91%	86.01%	63.34%	79.83	41.58	185.96
YOLO-ODS	85.69%	86.79%	86.24%	90.19%	67.20%	5.81	2.86	10.19

RT-DETR-ResNet50-Seg was constructed by integrating an instance segmentation head into the original RT-DETR-ResNet50 detection framework.

**Table 8:** Comparison with representative instance segmentation models on category-level instance segmentation performance.

Model	Canopy		Leaf vein	
	Mask mAP@0.5	Mask mAP@0.5:0.95	Mask mAP@0.5	Mask mAP@0.5:0.95
YOLOv5n-Seg	92.08%	79.95%	84.11%	49.32%
YOLOv6n-Seg	93.41%	81.06%	84.48%	48.89%
YOLOv8n-Seg	92.59%	80.39%	84.17%	49.36%
YOLOv10n-Seg	93.01%	80.27%	84.50%	49.01%
YOLOv11n-Seg	93.46%	81.11%	84.63%	49.08%
YOLOv12n-Seg	93.48%	81.18%	84.39%	49.26%
YOLOv26n-Seg	92.89%	81.68%	84.77%	49.05%
RT-DETR-ResNet50-Seg	91.91%	79.20%	80.12%	47.47%
YOLO-ODS	94.60%	82.91%	85.78%	51.49%

RT-DETR-ResNet50-Seg was constructed by integrating an instance segmentation head into the original RT-DETR-ResNet50 detection framework.

All comparison models were implemented based on publicly available open-source frameworks from Ultralytics (https://docs.ultralytics.com/) and their corresponding official releases. To ensure a fair comparison, all models were trained and evaluated under identical experimental conditions, including the same dataset partition, input image resolution, training epochs, optimizer, learning rate, batch size, data augmentation strategy, and evaluation metrics. For YOLO-based models with official instance segmentation implementations, the corresponding segmentation architectures were directly adopted. For models without directly available segmentation versions in the adopted framework, including YOLOv6n, YOLOv10n, and YOLOv12n, the original backbone and neck structures were retained, and a unified lightweight instance segmentation head was introduced for mask prediction. This design ensured that the comparison mainly reflected differences in feature extraction and multi-scale representation capability rather than variations caused by different segmentation heads. RT-DETR was originally designed as an end-to-end object detection framework (Zhao et al., 2024). Therefore, in this study, RT-DETR-ResNet50-Seg was constructed by retaining the original backbone, hybrid encoder, and decoder structures, while integrating an instance segmentation head consistent with the YOLO-based comparison models.

As shown in **Table 7**, YOLO-ODS achieved the best overall segmentation performance among all compared models. The proposed model obtained a Precision of 85.69%, Recall of 86.79%, F1-score of 86.24%, mask mAP@0.5 of 90.19%, and mask mAP@0.5:0.95 of 67.20%. Compared with the baseline YOLOv11n-Seg model, YOLO-ODS improved Recall, mask mAP@0.5, and mask mAP@0.5:0.95 by 2.20, 1.14, and 2.10 percentage points, respectively. These improvements demonstrate that the proposed OACM, DIAM, and Slide Loss effectively enhance feature representation and mask prediction quality for tobacco canopy and leaf vein segmentation.

Among the lightweight YOLO-based models, the overall performance differences were relatively small, with mask mAP@0.5:0.95 values ranging from 64.64% to 65.37%. This indicates that directly upgrading the backbone architecture or replacing the baseline model structure provides limited improvement for this specific task. In contrast, YOLO-ODS achieved a higher mask mAP@0.5:0.95 of 67.20%, demonstrating that incorporating directional feature modeling, attention-based feature refinement, and adaptive loss optimization is more effective for representing tobacco structures, especially slender leaf vein targets.

The Transformer-based RT-DETR-ResNet50-Seg achieved a mask mAP@0.5 and mask mAP@0.5:0.95 of 86.01% and 63.34%, respectively, which were lower than those of YOLO-ODS. Although Transformer architectures provide strong global feature modeling capability, RT-DETR-ResNet50-Seg required substantially higher computational resources, with 41.58 M parameters and 185.96 GFLOPs. In comparison, YOLO-ODS contained only 2.86 M parameters and required 10.19 GFLOPs. These results indicate that, in the present experiment, YOLO-ODS achieved a better accuracy-complexity trade-off than the evaluated RT-DETR-R50-based segmentation variant for UAV-based tobacco canopy and leaf vein instance segmentation, making it more suitable for deployment on resource-constrained platforms such as UAV edge devices and agricultural robots.

The category-level segmentation results are presented in **Table 8**. YOLO-ODS achieved strong performance for both canopy and leaf vein categories. For canopy segmentation, YOLO-ODS achieved mask mAP@0.5 and mask mAP@0.5:0.95 values of 94.60% and 82.91%, respectively, indicating its strong capability in recognizing large-scale canopy regions. Compared with other lightweight segmentation models, YOLO-ODS achieved the highest canopy mask mAP@0.5, demonstrating improved discrimination of tobacco plant instances under complex field conditions.

For leaf vein segmentation, which represents a more challenging fine-grained segmentation task, YOLO-ODS achieved mask mAP@0.5 and mask mAP@0.5:0.95 values of 85.78% and 51.49%, respectively. Compared with YOLOv11n-Seg, YOLO-ODS improved leaf vein mask mAP@0.5 and mask mAP@0.5:0.95 by 1.15 and 2.41 percentage points, respectively. The improvement in mask mAP@0.5:0.95 is particularly important because it indicates improved mask overlap and shape delineation under stricter IoU criteria of leaf vein structures, which directly affects the subsequent skeleton extraction and topology-guided stem point localization.

In terms of model complexity, YOLO-ODS contained 2.86 M parameters and required 10.19 GFLOPs, representing only slight increases compared with YOLOv11n-Seg (2.84 M parameters and 9.73 GFLOPs). Meanwhile, YOLO-ODS achieved substantially improved segmentation accuracy, especially for leaf vein structures. This demonstrates that the proposed modules enhance model representation ability while maintaining the lightweight characteristics of the baseline network.

Overall, YOLO-ODS outperformed the compared instance segmentation models in key indicators, including mask Recall, mask F1-score, mask mAP@0.5, and mask mAP@0.5:0.95, while maintaining low computational complexity. The comparison results verify the effectiveness of the proposed method for tobacco canopy and leaf vein instance segmentation under complex UAV field environments.

### 3.6 Stem point localization results and analysis

#### 3.6.1 Effect of proximal skeleton ratio on stem point localization

The proximal skeleton ratio has a substantial influence on the localization accuracy of the proposed topology-guided stem point localization method. If the selected proximal portion is too short, the fitted trend line may be unstable because only a limited number of skeleton points are used. In contrast, if the selected portion is too long, highly curved distal vein regions may be included, which can introduce directional bias and weaken the representation of the vein orientation near the stem region.

To evaluate the effect of this parameter, the proximal skeleton ratio was varied from 10% to 100% with an interval of 10%. A total of 100 test images were selected for parameter sensitivity analysis. From these images, tobacco plants with visible and manually identifiable upper stem regions were annotated, and the centroid of the visible upper stem region was used as the ground-truth stem point. The proposed topology-guided stem point localization method was then applied under different proximal skeleton ratios, and *MAE* and *RMSE* were used as evaluation metrics. The results are shown in **Table 9**.

**Table 9:** Effect of proximal skeleton ratio on stem point localization performance.

Proximal skeleton ratio	MAE /px	RMSE /px
10%	16.97	19.21
20%	13.58	14.68
30%	11.95	12.87
40%	12.45	13.67
50%	13.25	14.19
60%	12.43	13.60
70%	14.06	15.32
80%	15.83	17.60
90%	16.06	17.67
100%	15.86	16.89

As shown in **Table 9**, the localization error first decreases and then increases as the proximal skeleton ratio increases. When the ratio is 10%, the *MAE* and *RMSE* are 16.97 px and 19.21 px, respectively, indicating that too few skeleton points cannot provide a stable local direction estimate. When the ratio increases to 30%, the method achieves the best performance, with an *MAE* of 11.95 px and an *RMSE* of 12.87 px. When the ratio continues to increase beyond 30%, the localization accuracy gradually decreases because more distal skeleton points are included, and the curvature of distal vein regions introduces bias into the fitted trend line. Therefore, 30% is selected as the proximal skeleton ratio in the proposed method. This setting provides the best balance between directional stability and robustness to distal vein curvature.

#### 3.6.2 Error-propagation analysis results

To investigate the combined downstream influence of segmentation-mask quality and the residual limitations of geometric inference, three canopy and leaf vein mask conditions were compared while all subsequent processing steps were kept unchanged. The quantitative results are presented in **Table 10**, and representative localization cases are shown in **Figure 9**.

**Table 10:** Effect of segmentation-mask source on stem point localization performance.

Mask condition	MAE /px	RMSE /px	E_med_ /px	E_max_ /px	SR (10)	SR (20)	SR (30)	SLR / (%)
Ground-truth masks+proposed geometric inference	7.84	8.12	7.34	11.56	46.32%	100.00%	100.00%	97.65%
YOLO-ODS masks+proposed geometric inference	12.31	13.24	11.54	27.82	40.53%	88.95%	100.00%	94.84%
YOLOv11n-Seg masks+proposed geometric inference	13.82	14.12	13.44	31.00	38.95%	87.37%	98.42%	92.49%

The successful localization rate was calculated using all 213 tobacco plants. Localization error metrics were calculated using the same 190 tobacco plants that were successfully localized under all three mask conditions.

**Figure 9:**
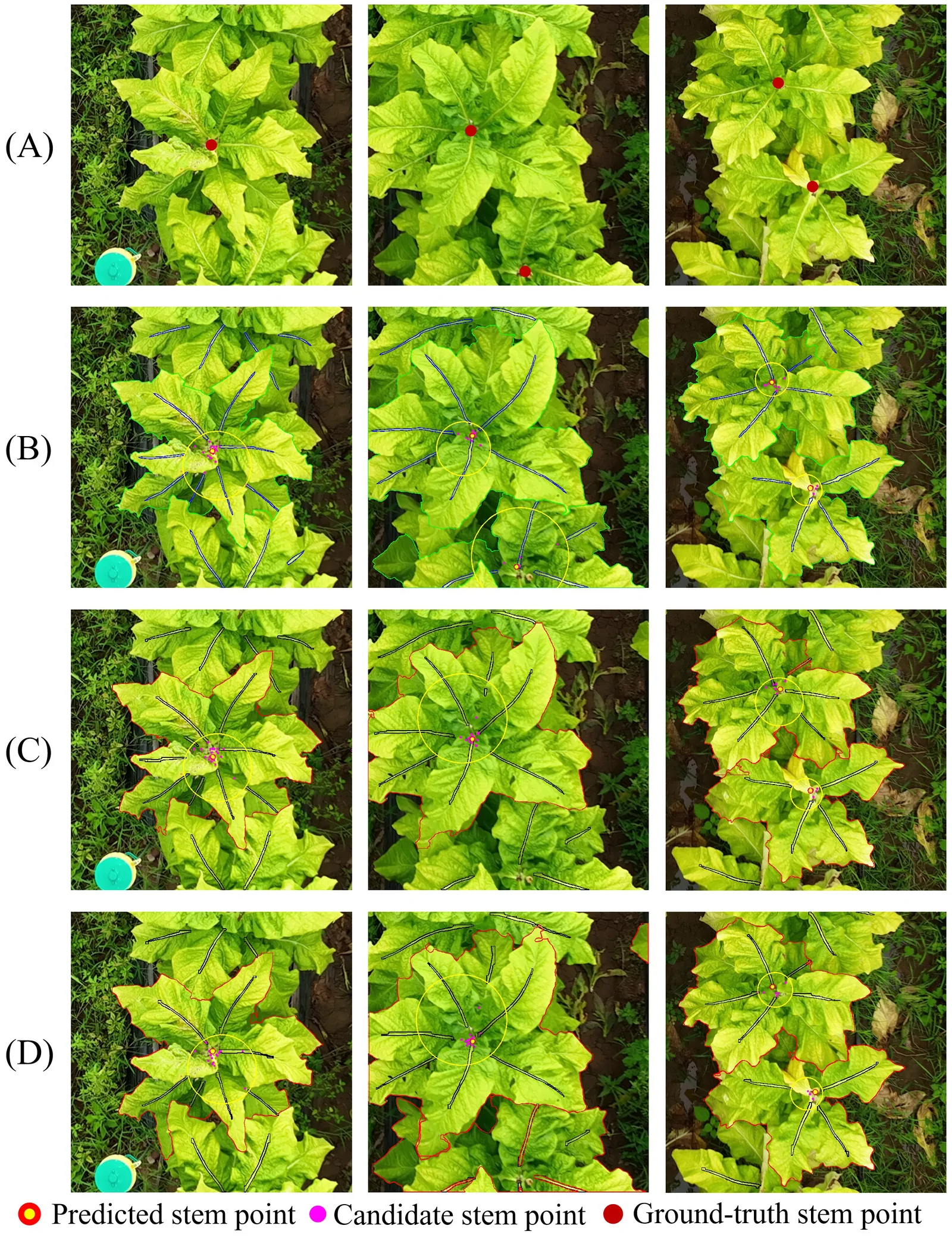
Stem point localization results under three canopy and leaf vein mask sources. **(A)** Original image; **(B)** Ground-truth masks+proposed geometric inference; **(C)** YOLO-ODS masks+proposed geometric inference; and **(D)** YOLOv11n-Seg masks+proposed geometric inference.

When manually annotated ground-truth masks were used, the proposed geometric inference procedure achieved an *MAE* of 7.84 px, an *RMSE* of 8.12 px, an *E*_med_ of 7.34 px, and an *E*_max_ of 11.56 px. The *SLR* reached 97.65%. Because prediction errors from the instance segmentation model were excluded under this condition, the remaining localization error primarily reflected limitations associated with the downstream processing procedure and the available structural information. These limitations included curvature of the proximal leaf vein regions, changes introduced during mask refinement and skeletonization, uncertainty in endpoint identification, deviations in local direction fitting, uneven spatial distributions of visible veins, and insufficient structural observability. The ground-truth-mask condition therefore provides an empirical reference for the residual localization error of the geometric pipeline under the current dataset, annotation protocol, and parameter settings, rather than a strict theoretical lower bound.

When YOLO-ODS-predicted masks were used, the *SLR* decreased from 97.65% to 94.84%, corresponding to a reduction of 2.81 percentage points. On the 190 plants successfully localized under all three conditions, the *MAE* increased from 7.84 px to 12.31 px, while the *RMSE* increased from 8.12 px to 13.24 px. The *E*_med_ and *E*_max_ increased to 11.54 px and 27.82 px, respectively. Because the downstream processing procedure was identical, these differences represent the additional localization degradation associated with the use of model-predicted canopy and leaf vein masks. When YOLOv11n-Seg-predicted masks were used, the SLR decreased further to 92.49%, while the *MAE* and *RMSE* increased to 13.82 px and 14.12 px, respectively. Compared with the YOLOv11n-Seg condition, the YOLO-ODS condition improved SLR by 2.35 percentage points and reduced *MAE* and *RMSE* by 1.51 px and 0.88 px, respectively. It also reduced *E*_med_ from 13.44 px to 11.54 px and *E*_max_ from 31.00 px to 27.82 px. These results indicate that the improvements in canopy and leaf vein segmentation achieved by YOLO-ODS were associated with improved downstream localization performance under the controlled processing conditions. More complete leaf vein masks provided a larger number of valid structural constraints, while improved canopy separation supported more reliable canopy-vein association and candidate-point filtering.

Segmentation-related errors affected stem point localization through three principal pathways. First, missed, fragmented, or incomplete leaf vein masks degraded the available topological information by reducing the number and continuity of valid skeletons. This could prevent the generation of sufficient trend-line intersections. Second, inaccuracies in leaf vein boundaries altered skeleton geometry and proximal endpoint positions, introducing deviations into local direction estimation. Even relatively small directional deviations could produce larger intersection displacements after trend-line extension. Third, incomplete canopy masks or confusion between adjacent canopy instances affected canopy-leaf vein association and spatial constraint filtering. Consequently, valid veins could be assigned to an incorrect canopy, or valid candidate intersections could be incorrectly removed. Segmentation errors therefore affected both the probability of producing a valid localization result and the accuracy of the predicted coordinate after successful localization.

The remaining localization failures were grouped into two categories. The first category was associated with insufficient observable structural information and occurred even under the ground-truth-mask condition. Severe canopy overlap sometimes left fewer than two visible and valid primary leaf veins, preventing pairwise trend-line intersection, as shown in **Figure 10A**. In other cases, the available veins were unevenly distributed or only partially visible, causing all candidate intersections to fall outside the canopy polygon or the minimum enclosing circle of the proximal endpoints, as shown in **Figure 10B**. The second category was associated with errors in model-predicted masks. Missed or fragmented leaf vein instances produced incomplete skeletons and unreliable local directions, preventing valid candidate intersections from being generated or retained, as shown in **Figure 10C**. Incomplete canopy segmentation and confusion between overlapping canopy instances affected canopy-leaf vein association and candidate-point filtering, as shown in **Figure 10D**.

**Figure 10:**
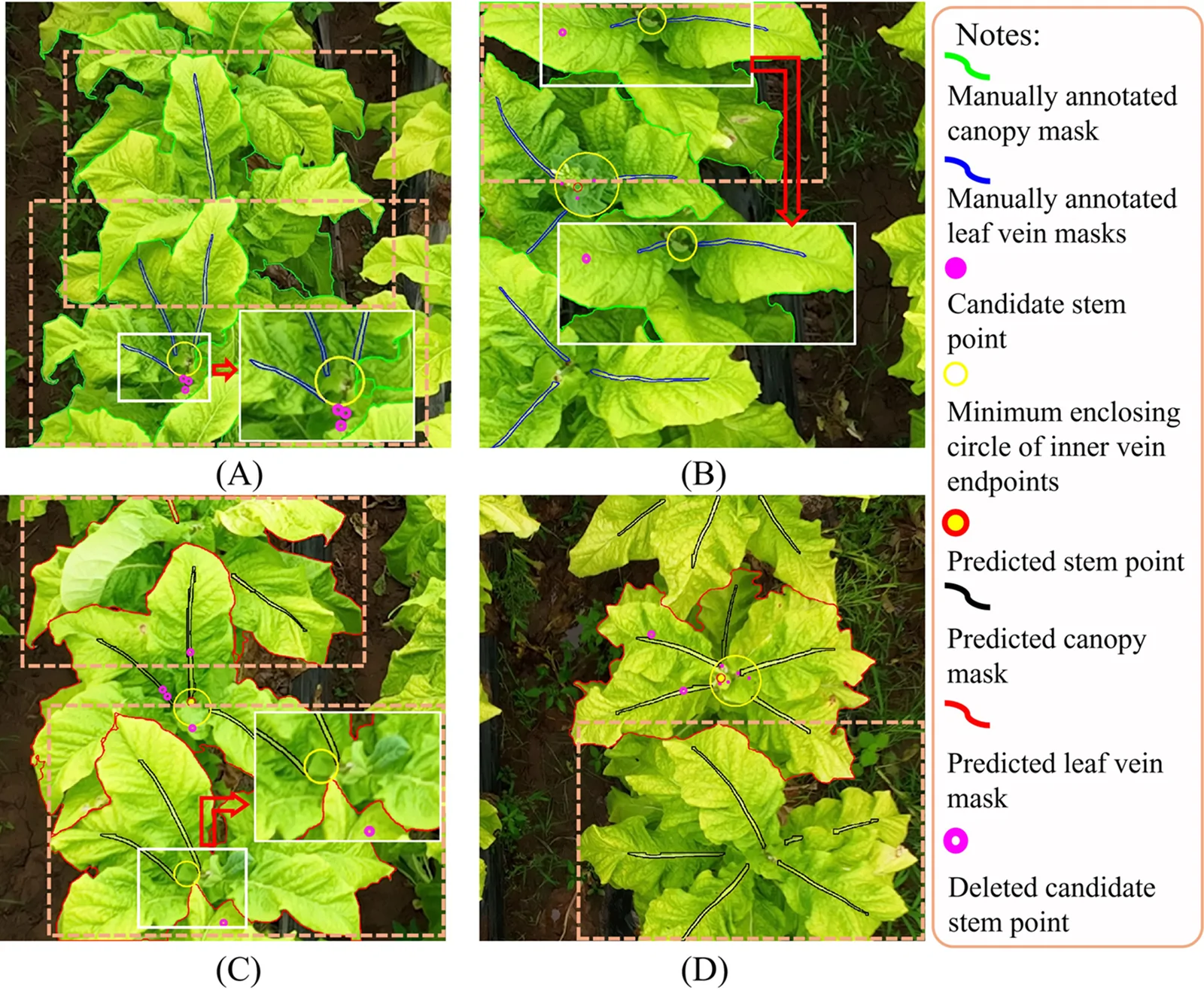
Representative failure cases and error sources in the segmentation-to-localization pipeline. **(A)** Insufficient observable leaf veins under the ground-truth-mask condition; **(B)** candidate intersections rejected by the spatial constraints under the ground-truth-mask condition; **(C)** leaf vein segmentation errors affecting skeleton extraction and local direction fitting; and **(D)** canopy segmentation errors affecting canopy-leaf vein association and candidate-point filtering.

Overall, the controlled analysis shows that the downstream geometric inference procedure can achieve relatively low localization error when reliable canopy and leaf vein structures are available. However, the nonzero error and localization failures observed under the ground-truth-mask condition also indicate that segmentation is not the only source of uncertainty. Structural observability, skeleton processing, local linear approximation, and spatial constraints impose an additional performance limit. The comparison between YOLO-ODS and YOLOv11n-Seg further indicates that improving the completeness, continuity, and instance consistency of canopy and primary leaf vein masks can improve both localization feasibility and conditional localization accuracy.

#### 3.6.3 Performance comparison of stem point localization methods

To evaluate the effectiveness of the proposed topology-guided stem point localization method, 70 tobacco plants were selected from low-altitude nadir-view UAV images acquired at a flight height of 6 m. Among them, 50 plants that were successfully localized by all four methods were selected and retained for the conditional localization-accuracy comparison.

For each plant, stem point coordinates predicted by four different localization strategies were obtained. Manually annotated stem point coordinates were used as ground truth for evaluation. The four compared strategies included a keypoint-regression-based method (YOLOv11n-pose), a bounding-box-center-based method, a canopy-centroid-based method, and the proposed topology-guided method. Quantitative results are presented in **Table 11**, and representative visualization examples are shown in **Figure 11**.

**Table 11:** Comparison of different stem point localization methods.

Method	MAE /px	RMSE /px	E_med_ /px	E_max_ /px	SR (10)	SR (20)	SR (30)	SLR / (%)
Keypoint-regression-based method	24.68	27.43	22.08	78.23	6.00%	34.00%	78.00%	91.43%
Bounding-box-center-based method	86.64	112.06	59.49	440.17	0.00%	6.00%	10.00%	95.71%
Canopy-centroid-based method	68.24	94.39	56.71	471.33	2.00%	6.00%	22.00%	95.71%
Proposed method	12.05	12.99	11.18	26.00	40.00%	92.00%	100.00%	92.86%

Note: The successful localization rate was calculated using all 70 tobacco plants. Localization error metrics were calculated using 50 successfully localized plants.

**Figure 11:**
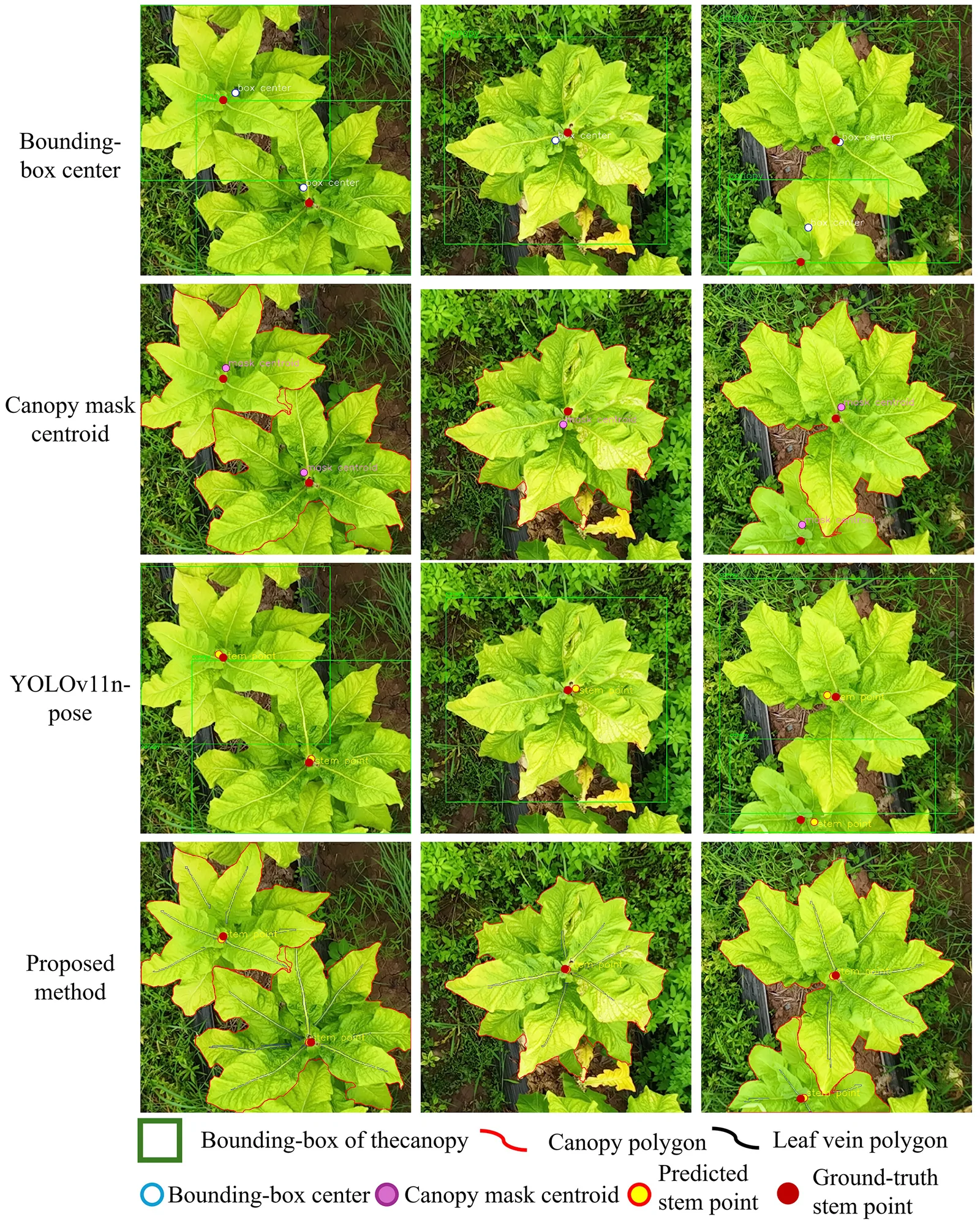
Visual comparison of stem point localization results obtained by different methods.

As shown in **Table 11**, regarding localization feasibility, the bounding-box-center-based and canopy-centroid-based methods achieved the highest successful localization rates, both reaching 95.71%, followed by the proposed topology-guided method at 92.86% and the keypoint-regression-based method at 91.43%. The relatively high successful localization rates of the two geometric-center-based methods can be attributed to their simple prediction mechanisms: once a canopy instance is detected or segmented, its bounding-box center or mask centroid can be directly generated as the stem point estimate. However, a high successful localization rate does not necessarily indicate high localization accuracy, as these geometric centers may deviate substantially from the actual stem position. In contrast, the proposed method requires sufficient valid leaf vein structures and candidate intersections satisfying the predefined spatial constraints. Although these requirements slightly reduce its successful localization rate, they substantially improve the accuracy and reliability of the generated stem point coordinates. Therefore, the SLR should be interpreted together with the localization error metrics rather than used independently to assess overall localization performance.

The proposed topology-guided method achieved the best localization performance among all compared methods. Its *MAE* and *RMSE* were 12.05 px and 12.99 px, respectively, and the *E*_med_ was 11.18 px. Compared with YOLOv11n-pose, whose *MAE* and *RMSE* were 24.68 px and 27.43 px, respectively, the proposed method reduced the *MAE* and *RMSE* by approximately 51.2% and 52.6%. This result indicates that directly regressing stem points from image appearance is less effective when the stem point is small, weakly textured, and visually similar to surrounding canopy tissues. In contrast, the proposed method uses the directional convergence of leaf veins as an additional geometric cue, thereby reducing the dependence on local stem appearance alone.

The two geometry-based approximation methods showed substantially lower localization accuracy. The bounding-box-center-based method and the canopy-centroid-based method achieved *MAE* values of 86.64 px and 68.24 px, respectively. Their *RMSE* values also exceeded 90 px. These large deviations mainly resulted from the asymmetric morphology of tobacco canopies caused by leaf extension, plant inclination, and overlapping neighboring plants. Consequently, the geometric center of the canopy region cannot reliably represent the actual stem position. The robustness advantage of the proposed method was further demonstrated by the *E*_max_. The proposed method achieved a *E*_max_ of only 26.00 px, whereas the bounding-box-center and canopy-centroid methods produced extremely large deviations of 440.17 px and 471.33 px, respectively. This indicates that the topology-guided strategy effectively suppresses abnormal localization failures caused by canopy deformation and adjacent-plant interference. The localization success rates under different pixel thresholds also demonstrate the superiority of the proposed method. The proposed method achieved *SR*(10), *SR*(20), and *SR*(30) values of 40.00%, 92.00%, and 100.00%, respectively, meaning that all 50 commonly localized plants had localization errors within 30 px. In comparison, the keypoint-regression-based method achieved *SR*(10), *SR*(20), and *SR*(30) values of only 6.00%, 34.00%, and 78.00%, respectively. The two geometric-center-based methods performed considerably worse, particularly under strict localization thresholds.

Under the nominal GSD calculated from the flight altitude and camera specifications, the *MAE* of 12.05 pixels and *RMSE* of 12.99 pixels obtained by the proposed localization method corresponded to approximately 1.94 cm and 2.09 cm, respectively. These results indicate that the proposed topology-guided localization method provides a potential spatial reference under low-altitude UAV imaging conditions for subsequent plant-level precision management and robotic operation.

### 3.7 Discussion

#### 3.7.1 Dataset limitations and external generalizability

The dataset used in this study was collected from two tobacco production fields in Guangdong Province under sunny, cloudy, and overcast conditions. Dataset partitioning was performed at the source-image level before patch extraction, thereby reducing the risk that patches originating from the same UAV image or overlapping field scene were distributed across the training, validation, and test subsets. In addition, a cross-location-and-date evaluation was conducted, and the corresponding results are provided in the **Supplementary Material**. These measures provide preliminary evidence that the proposed framework can maintain stable performance under moderate variations in illumination, acquisition date, and field location within the investigated production environments.

Nevertheless, the present results should not be interpreted as comprehensive evidence of external generalizability. Both acquisition sites were located in the same province, the dataset contained only the Yunyan 87 tobacco variety, and image acquisition was restricted to a relatively short period. Moreover, the stem point localization experiments primarily focused on topped tobacco plants for which the visible upper stem region could be reliably annotated. The current dataset may therefore not fully represent variations associated with tobacco variety, phenological stage, cultivation practice, canopy architecture, regional climate, soil background, or imaging platform.

The annotation scope represents an additional limitation of the current dataset. Only visible primary leaf veins were annotated, whereas secondary veins, severely occluded veins, and unclear or disconnected vein fragments were excluded. This strategy was adopted because primary leaf veins generally preserve a more direct and stable directional relationship with the stem region and are therefore more suitable for the convergence-based geometric inference used in the present framework. However, restricting the annotations to primary veins inevitably reduces the amount of vascular structural information available to both the segmentation model and the subsequent topology-guided localization procedure. Under real field conditions, primary veins may be partially occluded by overlapping leaves, fragmented because of weak image contrast, or incompletely recovered by the segmentation model. In such cases, secondary veins that remain visible cannot currently be exploited as auxiliary structural information.

Secondary veins may nevertheless provide useful cues when primary-vein information is incomplete. Because secondary veins branch from primary veins, their spatial arrangement and local orientations may assist in estimating the continuity or local direction of an occluded or fragmented primary vein. However, secondary veins cannot simply be treated as additional stem-convergence lines in the same manner as primary veins. Unlike primary veins, their directions do not necessarily point directly toward the stem region, and directly extending secondary-vein trajectories could introduce erroneous candidate intersections. Therefore, their potential value lies primarily in providing auxiliary topological information rather than independent stem-point constraints.

Future dataset development should therefore consider hierarchical annotation of the tobacco vascular structure by explicitly distinguishing primary and secondary veins and modeling their parent-child topological relationships. Under such a representation, secondary veins could potentially support the reconstruction of partially occluded primary veins, refine local primary-vein direction estimates, or provide confidence information for primary-vein trajectories. This may improve the structural observability and robustness of topology-guided stem localization under severe occlusion or vein fragmentation. At the same time, future studies should expand the dataset to include multiple tobacco varieties, growth and topping stages, planting seasons, and geographically independent test sites. External validation using different UAV platforms, cameras, flight heights, and ground sampling distances will also be necessary to determine whether the segmentation model and topology-guided localization parameters remain transferable without retraining or recalibration. Reporting performance separately according to acquisition site, date, illumination condition, growth stage, and planting density would further clarify the conditions under which performance degradation occurs.

#### 3.7.2 Technical origins and innovation boundaries of YOLO-ODS

The three components incorporated into YOLO-ODS originate from different methodological backgrounds and should therefore be understood as task-oriented adaptations and integrations of existing methodological concepts rather than three completely new fundamental theories proposed in this study. Their respective technical origins and contributions are distinguished below to clarify the innovation boundary of the proposed model.

OACM was designed in this study to address the strong directional characteristics of tobacco primary leaf veins and other elongated structural features. Its underlying motivation is related to established multi-branch and orientation-sensitive convolutional feature extraction approaches, where convolutional operations with different receptive-field orientations are employed to enhance the representation of anisotropic structures. Therefore, this study does not claim directional or multi-branch convolution itself as a fundamentally new concept. Instead, the contribution of OACM lies in its task-specific adaptation, including the combination of horizontal, vertical, 45°, and 135°feature extraction branches, direction-biased initialization for diagonal branches, spatially adaptive directional weighting, channel fusion, and residual learning. These components were integrated to improve the representation of slender and multi-directional structures in low-altitude UAV imagery while maintaining limited computational overhead. Thus, the novelty of OACM is reflected in its task-oriented design rather than the invention of a new convolution operation.

Similarly, DIAM builds upon established spatial and channel attention mechanisms. Spatial attention is generally used to identify informative spatial regions, whereas channel attention emphasizes semantically important feature channels. Therefore, the contribution of DIAM does not lie in proposing spatial or channel attention as new mechanisms. Instead, DIAM was designed as a task-oriented parallel spatial-channel recalibration module for the simultaneous segmentation of two structurally different tobacco targets: large and spatially continuous canopy regions and slender, low-contrast primary leaf veins. By generating spatial and channel attention representations in parallel and jointly applying them to the input feature representation, DIAM aims to preserve fine spatial information while improving semantic discrimination under complex field backgrounds.

In contrast to OACM and DIAM, Slide Loss is not an original methodological component introduced in this study. Slide Loss was proposed by Yu et al. (2024) as an adaptive sample-weighting strategy and was adopted here to adjust optimization emphasis among samples with different matching qualities. Therefore, its role in YOLO-ODS is the application and integration of an existing optimization strategy rather than the introduction of a new loss formulation. The present study evaluates the suitability of Slide Loss for tobacco canopy and primary leaf vein segmentation and analyzes its interaction with the proposed feature-representation modules.

Accordingly, the innovation of YOLO-ODS should be interpreted at the level of task-oriented methodological adaptation, module integration, and application-specific optimization rather than the introduction of three entirely new underlying concepts. Specifically, the contribution of this study lies in designing task-specific adaptations of OACM and DIAM for tobacco canopy and primary leaf vein characteristics, integrating these components with the existing Slide Loss strategy within the YOLOv11n-Seg framework, and systematically evaluating their individual and combined effects on the target segmentation task. More broadly, these improvements provide more reliable structural segmentation information for the subsequent topology-guided stem point localization framework. This definition more precisely delineates the methodological contribution of YOLO-ODS and avoids overstating the novelty of the individual underlying concepts.

#### 3.7.3 Biological rationale and trade-offs among localization strategies

The proposed method is based on the biological relationship between the tobacco stem and the primary leaf veins. Tobacco leaves originate from nodes along the stem and extend outward, while their primary veins generally preserve the dominant growth directions of the leaves. Under nadir-view imaging, the inward extensions of several visible primary veins therefore tend to converge near the upper stem region. This relationship provides a biologically meaningful structural cue for localizing a stem point that is itself small, weakly textured, and difficult to distinguish from the surrounding canopy tissues.

The observed performance differences among the evaluated localization strategies are consistent with the advantages expected from this structural prior. Appearance-based keypoint regression directly estimates stem coordinates from image features and is therefore sensitive to the limited visual saliency of the visible upper stem. Bounding-box-center and canopy-centroid methods are computationally simple and can return a coordinate whenever a canopy is detected or segmented. However, these methods implicitly assume that the stem lies close to the geometric center of the canopy. This assumption is frequently violated by asymmetric leaf expansion, plant inclination, incomplete canopy visibility, and overlap between neighboring plants.

In contrast, the proposed method integrates multiple leaf vein directions rather than relying on a single appearance feature or geometric-center estimate. On the commonly localized samples, it achieved lower conditional *MAE*, *RMSE*, and maximum localization error than the keypoint-regression, bounding-box-center, and canopy-centroid methods. The reduced maximum error also indicates that the topology-guided strategy was less susceptible to extreme deviations caused by asymmetric canopy geometry.

The improvement in localization accuracy was accompanied by a trade-off in localization feasibility. The bounding-box-center and canopy-centroid methods can generate a coordinate once a valid canopy instance is available, whereas the proposed method requires at least two valid primary leaf veins and candidate intersections satisfying the predefined spatial constraints. Consequently, its successful localization rate was slightly lower than those of the two geometric-center methods, despite its substantially lower conditional localization errors. Successful localization rate and localization accuracy should therefore be interpreted jointly. A method that always returns a coordinate is not necessarily accurate, whereas a structurally constrained method may reject cases with insufficient evidence rather than output a highly uncertain prediction.

The biological prior is conditional rather than universally applicable. Reliable topology-guided inference requires multiple observable primary veins, proximal vein directions that approximately point toward the stem region, and correct association of the veins with the corresponding canopy. Strong vein curvature, leaf twisting, severe occlusion, canopy overlap, or incorrect canopy-leaf vein association may violate these assumptions. The proposed approach should therefore be regarded as a biologically informed localization strategy with explicit applicability requirements rather than a universal replacement for appearance-based localization methods.

#### 3.7.4 Practical applicability and failure modes under field conditions

The proposed framework may fail to generate a valid stem point prediction when sufficient structural information is unavailable. The error-propagation analysis showed that localization failures may arise from limited leaf vein observability, segmentation errors, or instability in geometric inference. When fewer than two valid primary veins are visible, trend-line intersections cannot be constructed. Fragmented vein masks, inaccurate skeletons, incorrect canopy-leaf vein associations, or nearly parallel trend lines may also prevent valid candidate points from being generated or retained.

Beyond these observed failure modes, several field-level factors may further increase the risk of localization failure. UAV flight height directly affects the effective spatial resolution of leaf vein structures. As flight height increases, primary veins are represented by fewer pixels, reducing their apparent width and contrast and potentially weakening vein segmentation, skeleton continuity, and local direction estimation. The proposed method is therefore more suitable for low-altitude UAV imagery in which primary leaf veins remain sufficiently resolved. Accordingly, the approximate ground-equivalent localization accuracy reported in this study should be interpreted within the evaluated imaging conditions and should not be generalized directly to substantially higher flight altitudes.

Plant growth stage also affects the reliability of the topology prior. The method was primarily developed for topped tobacco plants, in which the upper stem region and surrounding primary veins exhibit a relatively clear structural relationship. At earlier growth stages, the number, length, or directional stability of visible primary veins may be insufficient for reliable inference. At later stages, canopy closure, leaf bending, and inter-plant occlusion may reduce vein visibility and weaken the convergence consistency of the fitted directions.

Planting density mainly influences canopy separation, structural observability, and canopy-leaf vein association. Under high-density conditions, leaves from neighboring plants may overlap, causing adjacent canopy instances to merge or be incompletely segmented. Primary veins may consequently be assigned to incorrect canopies, while valid candidate intersections may be rejected by canopy-based spatial constraints. Dense planting may also reduce the number of unobstructed veins available for each plant, further increasing the probability of localization failure.

The effects of flight height, growth stage, planting density, and occlusion severity were not independently quantified in the present study. These factors should therefore be regarded as plausible practical limitations rather than experimentally established failure thresholds. Future studies should evaluate the framework under controlled variations in these conditions. Further improvements may include topology-aware leaf vein segmentation, uncertainty-weighted geometric inference, multi-view or temporal image fusion, and fallback localization strategies for plants with insufficient leaf vein information.

## 4 Conclusions

This study proposed a lightweight instance segmentation and topology-guided stem point localization framework for tobacco plants in low-altitude nadir-view UAV imagery. A YOLOv11n-Seg-based model, termed YOLO-ODS, was developed for joint segmentation of tobacco canopies and leaf veins. By integrating orientation-aware convolution, dual interactive attention, and Slide Loss, YOLO-ODS enhanced the representation of directional and fine-grained structures while maintaining low computational complexity. The model achieved a Recall of 86.79%, a mask mAP@0.5 of 90.19%, and a mask mAP@0.5:0.95 of 67.20%, demonstrating improved segmentation performance compared with the YOLOv11n-Seg baseline.

Based on the segmentation results, stem point localization was further formulated as a topology-guided geometric inference problem rather than a direct appearance-based detection task. leaf vein skeletons were extracted, local trend lines were fitted near the proximal vein regions, and candidate stem points were generated from the geometric convergence of leaf vein directions under canopy constraints. This strategy effectively exploited the morphological relationship between tobacco leaf veins and stem regions. In the localization experiment, the proposed method achieved an *MAE* of 12.05 px and an *RMSE* of 12.99 px, substantially outperforming the keypoint-regression-based, bounding-box-center-based, and canopy-centroid-based methods.

The results indicate that combining lightweight instance segmentation with explicit geometric priors can improve the localization of visually weak plant keypoints in complex field scenes. In particular, leaf vein convergence provides an interpretable and biologically meaningful cue for inferring stem point positions when the visible stem region is small, weakly textured, and visually similar to surrounding canopy tissues. Therefore, the proposed framework has potential applications in UAV-based intelligent tobacco production, including robotic harvesting, precision field management, and plant-level phenotyping. Its lightweight design also supports possible deployment on edge devices for field monitoring and intelligent agricultural operations.

Nevertheless, the proposed method still has several limitations. Its localization accuracy may be affected by strong canopy overlap between adjacent plants, incomplete leaf vein segmentation, weak vein visibility, and inaccurate skeleton extraction. In addition, the stem point localization experiment was conducted using a limited number of annotated samples. Future work will focus on expanding stem point annotations across different growth stages, flight heights, planting densities, and field conditions. Further improvements will also be made to enhance leaf vein extraction, candidate-point filtering, and topology-guided inference under more complex canopy structures.

## Data Availability

The original contributions presented in the study are included in the article/**Supplementary Material**. Further inquiries can be directed to the corresponding authors.
