# Supplementary material for: Canopy and leaf vein segmentation and topology-guided localization of stem points in tobacco plants using YOLO-ODS
Source: Front Plant Sci. 2026 Oct 7;17:1930225. doi: 10.3389/fpls.2026.1930225 (PMC13685701; doi:10.3389/fpls.2026.1930225)
Supplement: Supplementary file 1 [file Table1.docx]

Supplementary Material

# 1 Effect of Acquisition-Sequence-Based Dataset Partitioning on Model Performance

To evaluate whether the original source-image-level dataset partitioning strategy might allow visually similar images from the same UAV acquisition sequence to be distributed across different subsets, an additional experiment was conducted using an acquisition-sequence-based independent partitioning strategy. All original UAV images acquired during the same flight sequence, together with their derived image patches, were assigned exclusively to a single subset.

The detailed dataset partitioning scheme under this acquisition-sequence-based independent strategy is summarized in Supplementary Table S1. Compared with the original source-image-level partitioning strategy, this strategy provides a stricter evaluation setting by preventing image patches derived from the same UAV acquisition sequence from being distributed across different subsets, thereby reducing the potential influence of highly similar image content on model evaluation.

Supplementary Table S1. Acquisition-sequence-based independent dataset partitioning according to acquisition location and date.

| Subset | Location | Collection date | Number of image patches | Total |
| --- | --- | --- | --- | --- |
| Training | Jiaoling County | 26 May 2025 | 506 | 1799 |
| Training | Jiaoling County | 27 May 2025 | 415 |  |
| Training | Xingzi Town | 29 June 2025 | 441 |  |
| Training | Xingzi Town | 30 June 2025 | 437 |  |
| Validation | Xingzi Town | 1 July 2025 | 561 | 561 |
| Test | Jiaoling County | 25 May 2025 | 248 | 248 |

Using this acquisition-sequence-based independent partitioning strategy, the training set was augmented using the same data augmentation methods described in the main manuscript, resulting in 3,598 training images. The YOLO-ODS model was then retrained under experimental settings identical to those used for the original partitioning strategy, including the same network architecture, training parameters, and evaluation metrics. The segmentation performance obtained under the original source-image-level partitioning strategy and the acquisition-sequence-based independent partitioning strategy is compared in Supplementary Table S2.

Supplementary Table S2. Comparison of YOLO-ODS performance under different dataset partitioning strategies.

| Splitting strategy | Training images after augmentation | Validation | Test | Mask Precision | Mask Recall | Mask F1-score | Mask mAP@0.5 | Mask mAP@0.5:0.95 |
| --- | --- | --- | --- | --- | --- | --- | --- | --- |
| Original source-image-level partitioning | 3650 | 522 | 261 | 85.69% | 86.79% | 86.24% | 90.19% | 67.20% |
| Acquisition-sequence-based independent partitioning | 3598 | 561 | 248 | 86.01% | 86.36% | 86.18% | 90.31% | 67.17% |

The results showed that YOLO-ODS achieved highly consistent segmentation performance under the two dataset partitioning strategies. The differences between the two strategies were no greater than 0.43 percentage points across all evaluation metrics. In particular, under the stricter acquisition-sequence-based independent partitioning strategy, the model achieved mask mAP@0.5 and mask mAP@0.5:0.95 values of 90.31% and 67.17%, respectively, which were comparable to the corresponding values of 90.19% and 67.20% obtained under the original source-image-level partitioning strategy.

The comparable results obtained under the acquisition-sequence-based independent split suggest that the performance reported using the original source-image-level split was unlikely to have been substantially inflated by similarities among images originating from the same UAV acquisition sequence. These findings provide additional evidence that the model performance was robust to variations in acquisition date and UAV flight sequence within the investigated fields.

# 2 Ablation Analysis of Data Augmentation Strategies and Visualization Analysis

## 2.1 Ablation Analysis of Data Augmentation Strategies

Data augmentation was applied exclusively to the training subset, whereas the validation and test subsets remained unchanged. The parameter settings and sampling probabilities for brightness adjustment, Gaussian blur, image rotation, and Gaussian noise are provided in Table 2 of the main manuscript.

To quantify the contribution of each augmentation operation, additional ablation experiments were conducted using the same YOLO-ODS architecture and identical training settings. The original 1,825 training patches were used as the augmentation source. Following the augmentation procedure described in the main manuscript, one augmented counterpart was generated for each original image while retaining the original image, resulting in a total of 3,650 training samples.

Seven training strategies were compared:

(1) No augmentation: The model was trained using only the original 1,825 training images without any augmentation operation.

(2) Duplicate control: The training set was expanded to 3,650 images by duplicating the original training images without applying any image transformation. This strategy was designed to distinguish the effect of increased sample quantity from the effect of increased visual diversity.

(3) Full augmentation: The same augmented training set as that used in the manuscript was adopted, where brightness adjustment, Gaussian blur, Gaussian noise, and image rotation were randomly applied according to their predefined sampling probabilities.

(4) Brightness-only augmentation: Only brightness adjustment was applied.

(5) Gaussian blur-only augmentation: Only Gaussian blur was applied.

(6) Rotation-only augmentation: Only image rotation was applied.

(7) Gaussian noise-only augmentation: Only Gaussian noise was applied.

All models were trained using the same network architecture, optimizer, training parameters, dataset partition, and evaluation protocol. Segmentation performance was evaluated using mask Precision, mask Recall, mask F1-score, mask mAP@0.5, and mask mAP@0.5:0.95. The results are summarized in Supplementary Table S3.

Supplementary Table S3. Overall segmentation performance under different data augmentation strategies.

| Augmentation strategy | Training images | Mask Precision | Mask Recall | Mask F1-score | Mask mAP@0.5 | Mask mAP@0.5:0.95 |
| --- | --- | --- | --- | --- | --- | --- |
| No augmentation | 1825 | 84.35% | 88.12% | 86.19% | 89.91% | 66.42% |
| Duplicate control | 3650 | 84.07% | 88.38% | 86.17% | 90.40% | 66.40% |
| Full augmentation | 3650 | 85.69% | 86.79% | 86.24% | 90.19% | 67.20% |
| Brightness-only augmentation | 3650 | 87.45% | 85.27% | 86.35% | 89.44% | 66.58% |
| Gaussian blur-only augmentation | 3650 | 85.10% | 88.71% | 86.87% | 90.04% | 66.58% |
| Rotation-only augmentation | 3650 | 85.43% | 87.88% | 86.64% | 90.51% | 67.12% |
| Gaussian noise-only augmentation | 3650 | 85.81% | 87.19% | 86.49% | 90.56% | 67.15% |

As shown in Supplementary Table S3, simply duplicating the original training images did not produce a substantial improvement in segmentation performance. Compared with the no-augmentation strategy, the duplicate-control strategy increased mask mAP@0.5 from 89.91% to 90.40%, whereas mask mAP@0.5:0.95 remained nearly unchanged, decreasing slightly from 66.42% to 66.40%. This result suggests that repeated exposure to the same images provides limited benefit under stricter mask-overlap criteria and that visual diversity is more important for improving fine-grained segmentation performance.

The individual augmentation operations affected different performance metrics. Brightness-only augmentation achieved the highest mask Precision of 87.45%, suggesting that simulated illumination variation improved the model’s ability to distinguish tobacco structures from complex field backgrounds. Gaussian blur-only augmentation produced the highest mask Recall and mask F1-score, reaching 88.71% and 86.87%, respectively, indicating improved tolerance to mild defocus and reduced image sharpness. Rotation-only and Gaussian noise-only augmentation achieved relatively high mask mAP@0.5 values of 90.51% and 90.56%, respectively, demonstrating their contributions to robustness against orientation changes and imaging disturbances. Although several individual augmentation strategies achieved higher mask mAP@0.5 values than the full augmentation strategy, the full augmentation strategy obtained the highest mask mAP@0.5:0.95 of 67.20%. Because mask mAP@0.5:0.95 evaluates segmentation performance across multiple, increasingly strict mask IoU thresholds, this result indicates that combining several augmentation operations improved the overall quality and consistency of predicted masks.

To further investigate category-specific effects, the segmentation performance of the canopy and leaf vein categories was analyzed separately. The results are presented in Supplementary Table S4.

Supplementary Table S4. Category-level segmentation performance under different data augmentation strategies.

| Augmentation strategy | Canopy | | Leaf vein | |
| --- | --- | --- | --- | --- |
|  | Mask mAP@0.5 | Mask mAP@0.5:0.95 | Mask mAP@0.5 | Mask mAP@0.5:0.95 |
| No augmentation | 94.81% | 81.75% | 85.01% | 51.08% |
| Duplicate control | 95.73% | 81.63% | 85.06% | 51.17% |
| Full augmentation | 94.60% | 82.91% | 85.78% | 51.49% |
| Brightness-only augmentation | 93.91% | 82.00% | 84.97% | 51.15% |
| Gaussian blur-only augmentation | 94.85% | 82.36% | 85.22% | 50.79% |
| Rotation-only augmentation | 95.78% | 83.19% | 85.23% | 51.04% |
| Gaussian noise-only augmentation | 95.82% | 83.23% | 85.29% | 51.07% |

As shown in Supplementary Table S4, the augmentation strategies produced different effects on canopy and leaf vein segmentation. For the canopy category, several individual augmentation strategies outperformed the full augmentation strategy. Gaussian noise-only augmentation achieved the highest canopy mask mAP@0.5 and mask mAP@0.5:0.95 values of 95.82% and 83.23%, respectively. Rotation-only augmentation achieved comparable canopy performance, indicating that large canopy structures benefited particularly from improved tolerance to spatial and imaging variations.

For the leaf vein category, however, the full augmentation strategy achieved the best performance, with mask mAP@0.5 and mask mAP@0.5:0.95 values of 85.78% and 51.49%, respectively. Compared with the no-augmentation strategy, full augmentation improved leaf vein mask mAP@0.5 and mask mAP@0.5:0.95 by 0.77 and 0.41 percentage points, respectively. Although these improvements were moderate, they were consistently observed under both evaluation criteria. Because leaf vein masks provide the structural basis for skeleton extraction and topology-guided stem point localization, improvements in leaf vein segmentation are particularly important for the complete framework.

Overall, the results demonstrate that different augmentation operations contribute complementary forms of robustness. Brightness adjustment improves adaptation to illumination variations, Gaussian blur enhances tolerance to reduced image quality, rotation improves robustness to orientation changes, and Gaussian noise increases resistance to imaging disturbances. The full augmentation strategy provides the most balanced improvement across disturbance types and achieves the best segmentation performance for the fine-scale leaf vein category.

## 2.2 Visualization Analysis of Different Augmentation Strategies

To further examine the influence of data augmentation on model robustness, qualitative visualization experiments were conducted. Supplementary Figure S1 compares the segmentation results produced by YOLO-ODS models trained using the seven augmentation strategies under representative brightness variation, Gaussian blur, rotation, and Gaussian noise conditions.

Models trained using individual augmentation operations generally demonstrated improved adaptation to their corresponding disturbance types. For example, the brightness-only model maintained relatively stable predictions under illumination changes, whereas the Gaussian blur-only model preserved more consistent segmentation results when image sharpness was reduced. Compared with the individual augmentation strategies, the model trained using full augmentation exhibited more balanced performance across the different disturbance conditions. It maintained relatively complete canopy boundaries and more continuous leaf vein predictions under diverse image variations, indicating improved robustness to combined field-imaging disturbances.

Based on the quantitative and qualitative results, the full augmentation strategy was adopted for the final YOLO-ODS model. Although individual augmentation strategies provided advantages for specific metrics or target categories, the combined strategy achieved the best mask mAP@0.5:0.95 and the strongest leaf vein segmentation performance. These characteristics are particularly important for the subsequent topology-guided stem point localization procedure.

Supplementary Figure S1. Qualitative comparison of YOLO-ODS models trained using different data augmentation strategies under four simulated image disturbances. Rows represent brightness variation, Gaussian blur, image rotation, and Gaussian noise, whereas columns represent: (A) no augmentation; (B) duplicate control; (C) full augmentation; (D) brightness-only augmentation; (E) Gaussian blur-only augmentation; (F) rotation-only augmentation; and (G) Gaussian noise-only augmentation.

# 3 Effect of Input Image Resolution on Segmentation Performance

The original UAV images were cropped into patches of 1280 × 1280 pixels during dataset construction, whereas the default input resolution used for YOLO-ODS training and inference was 640 × 640. Although reducing the input resolution decreases computational requirements, it may also reduce the representation capability for fine structures such as tobacco leaf veins.

To investigate the influence of input resolution and determine an appropriate resolution setting, additional experiments were conducted using input sizes of 640 × 640, 960 × 960, and 1280 × 1280. All experiments used the same dataset, model architecture, training and evaluation procedures, and were conducted under the same parameter settings, software environment, and hardware configuration as those adopted in the main experiments.

In addition to segmentation accuracy metrics, computational efficiency was evaluated using inference speed and peak GPU memory usage. Inference speed represents the number of images processed per second during model inference under identical hardware and software conditions. Peak GPU memory usage during inference represents the maximum GPU memory allocated during inference, reflecting the runtime memory requirement of different input resolutions. The experimental results are shown in Supplementary Table S5 and Supplementary Table S6.

Supplementary Table S5. Effect of input image resolution on overall instance segmentation performance and computational efficiency.

| Input size (pixels) | Mask Precision | Mask Recall | Mask F1-score | Mask mAP@0.5 | Mask mAP@0.5:0.95 | Model size (MB) | Parameters (M) | GFLOPs | Inference Speed /$\text{(frame}\text{∙}\text{s}^{\text{-1}}\text{)}$ | Peak GPU memory usage during inference (GB) |
| --- | --- | --- | --- | --- | --- | --- | --- | --- | --- | --- |
| 640 | 85.69% | 86.79% | 86.24% | 90.19% | 67.20% | 5.81 | 2.86 | 10.19 | 154.04 | 4.29 |
| 960 | 86.25% | 87.25% | 86.75% | 90.98% | 67.96% | 5.87 | 2.86 | 22.93 | 136.49 | 9.37 |
| 1280 | 85.34% | 86.37% | 85.85% | 89.88% | 65.02% | 5.96 | 2.86 | 40.76 | 96.46 | 15.86 |

As shown in Supplementary Table S5, the 960 × 960 input achieved the highest overall segmentation accuracy. Compared with the 640 × 640 setting, the 960 × 960 input improved mask Precision, mask Recall, mask F1-score, mask mAP@0.5, and mask mAP@0.5:0.95 by 0.56, 0.46, 0.51, 0.79, and 0.76 percentage points, respectively. This indicates that increasing the input resolution can preserve additional spatial information and slightly improve segmentation performance.

However, the performance improvement achieved by increasing the input resolution was accompanied by substantially increased computational and memory requirements. Compared with the 640 × 640 setting, the 960 × 960 input resolution increased the computational cost from 10.19 GFLOPs to 22.93 GFLOPs, while peak GPU memory usage increased from 4.29 GB to 9.37 GB. Meanwhile, the inference speed decreased from 154.04 to 136.49 $\text{frame}\text{∙}\text{s}^{\text{-1}}$.

When the input resolution was further increased to 1280 × 1280, the segmentation performance did not continue to improve. Instead, the mask mAP@0.5:0.95 decreased to 65.02%, while the computational cost and memory consumption further increased, with GFLOPs reaching 40.76 GFLOPs and peak GPU memory usage increasing to 15.86 GB. The inference speed also decreased to 96.46 $\text{frame}\text{∙}\text{s}^{\text{-1}}$. These results indicate that simply increasing input resolution does not necessarily lead to improved segmentation performance under the same network architecture and training strategy, while substantially increasing computational and memory requirements under the evaluated configuration.

The parameter count remained unchanged at 2.86 M because the network architecture was identical. The minor differences in checkpoint file size likely resulted from serialization and compression rather than changes in network architecture or parameter count. In contrast, GFLOPs and peak GPU memory usage increased substantially because larger input images generated larger intermediate feature maps.

Supplementary Table S6. Effect of input image resolution on category-level instance segmentation performance.

| Input size | canopy | | leaf vein | |
| --- | --- | --- | --- | --- |
|  | Mask mAP@0.5 | Mask mAP@0.5:0.95 | Mask mAP@0.5 | Mask mAP@0.5:0.95 |
| 640 | 94.60% | 82.91% | 85.78% | 51.49% |
| 960 | 95.64% | 83.74% | 86.31% | 52.18% |
| 1280 | 94.09% | 77.89% | 85.67% | 52.14% |

At the category level, the 960 × 960 input achieved the highest segmentation performance for both canopy and leaf vein categories. For canopy segmentation, mask mAP@0.5 and mask mAP@0.5:0.95 reached 95.64% and 83.74%, respectively. For leaf vein segmentation, mask mAP@0.5 and mask mAP@0.5:0.95 reached 86.31% and 52.18%, respectively. Compared with 640 × 640, the 960 × 960 resolution improved leaf vein mask mAP@0.5 and mask mAP@0.5:0.95 by 0.53 and 0.69 percentage points, respectively. This confirms that moderate resolution enhancement can preserve additional fine-grained vein information. However, at 1280 × 1280, although leaf vein mask mAP@0.5:0.95 remained slightly higher than that of 640 × 640, canopy mask mAP@0.5:0.95 decreased substantially to 77.89%, resulting in lower overall segmentation performance. This suggests that retaining more image details does not always translate into improved segmentation accuracy.

Considering segmentation accuracy, inference efficiency, and GPU memory requirements, 640 × 640 was retained as the default input resolution for YOLO-ODS. Compared with 960 × 960, the 640 × 640 setting reduced GFLOPs by 55.6% and peak GPU memory usage by 54.2%, while maintaining comparable segmentation performance. The overall mask mAP@0.5:0.95 and leaf vein mask mAP@0.5:0.95 decreased by only 0.76 and 0.69 percentage points, respectively. Considering segmentation accuracy and computational requirements under the evaluated GPU setting, 640 × 640 was retained as the default input resolution for subsequent experiments and deployment-oriented development.
